# Transition‐Metal‐Free Zeolite Composites for Tandem Catalytic Conversion of Methane to Light Olefins

**DOI:** 10.1002/advs.202515145

**Published:** 2025-10-13

**Authors:** Peipei Xiao, Hiroto Toyoda, Yuqin Sun, Yong Wang, Hermann Gies, Toshiyuki Yokoi

**Affiliations:** ^1^ Institute of Integrated Research Institute of Science Tokyo 4259 Nagatsuta, Midori‐ku Yokohama 226–8501 Japan; ^2^ iPEACE223 Inc. Konwa Building, 1‐12‐22 Tsukiji, Chuo‐ku Tokyo 104‐0045 Japan

**Keywords:** acidic zeolite, FER zeolite, methane oxidation, methanol to olefins, tandem catalysis

## Abstract

The methanol‐to‐olefins (MTO) reaction is considered one of the most important reactions in C1 chemistry, offering a route to produce basic petrochemicals from non‐oil resources such as natural gas and coal. Direct conversion of methane, the primary component of natural gas, to olefins via methanol as an intermediate is of significant industrial interest. Recent studies demonstrate that methanol can be efficiently synthesized from methane using transition‐metal‐free aluminosilicate Ferrierite (FER) zeolite with nitrous oxide (N_2_O) as the oxidant. Herein, a tandem catalytic system based on the composite FER and one more acidic zeolite is reported to achieve continuous conversion of methane to olefins. The topology and acidity of acidic zeolites critically influenced product distribution and hydrocarbon formation rates. When small‐pore zeolites are used as acidic zeolites, complete conversion of methanol to olefins is successfully achieved. Reaction conditions for methane‐to‐olefins conversion are optimized using  silicoaluminophosphate (SAPO‐34) as a representative acidic zeolite. The mass ratio of FER to SAPO‐34 determined catalytic performance, with conversion of methane to light olefins achieving thermodynamic feasibility at 325–400 °C. Enhanced intimacy between FER and SAPO‐34 particles promoted methane (CH_4_) conversion. This work establishes an efficient strategy for high‐selectivity light olefin production from methane over integrated transition‐metal‐free zeolite catalysts.

## Graphical abstract

Light olefins can be produced from methane using a cascade catalytic system that combines transition‐metal‐free (FER zeolite with an acidic zeolite, such as SAPO‐34, under relatively mild conditions. In this tandem process, methane is first oxidized to methanol over the FER zeolite and then converted to olefins via the MTO reaction on the acidic zeolite.

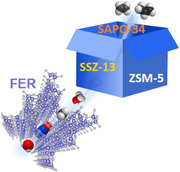

## 1 Introduction

Olefins such as ethylene (C_2_^=^) and propylene (C_3_^=^) are foundational building blocks in the chemical industry, essential for producing polymers, solvents, and fine chemicals.^[^ ^1^, ^2^, ^3^ ^]^ Traditionally, these compounds are derived from petroleum through energy‐intensive processes such as steam cracking, catalytic cracking, and hydrocracking.^[^ ^4^, ^5^, ^6^ ^]^ With the growing abundance of natural gas, methane has emerged as an attractive alternative feedstock.^[^ ^7^ ^]^ However, its direct conversion to olefins remains a formidable challenge due to the inertness of methane and its propensity toward over‐oxidation to carbon dioxide (CO_2_) under aggressive reaction conditions.^[^ ^8^, ^9^, ^10^ ^]^ Current industrial approaches often rely on indirect, multi‐step pathways, such as steam reforming to syngas, followed by the Fischer‐Tropsch synthesis, capital‐intensive and inefficient processes.^[^ ^9^ ^]^ Upgrading of methane to olefins (DMTO) represents a promising alternative but typically requires transition‐metal catalysts (e.g., Fe, Cu)^[^ ^8^, ^11^, ^12^, ^13^ ^]^ or noble‐metal catalysts^[^ ^14^, ^15^ ^]^ to activate methane. However, these catalysts suffer from sintering and raise environmental concerns about metal scarcity and toxicity.^[^ ^16^ ^]^ Moreover, achieving high olefin selectivity while suppressing CO_2_ formation remains a significant challenge. In the past research, zeolite systems combining noble or transition metals with Brønsted acid sites (BAS) have been applied in tandem for the initial oxidation of methane to methanol (DMTM), followed by the conversion of methanol to hydrocarbons (MTH).^[^ ^17^, ^18^, ^19^ ^]^ For example, Román‐Leshkov and co‐workers employed a tandem catalyst system comprising Cu‐SSZ‐13 and H‐ZSM‐5 zeolites for the direct partial oxidation of CH_4_ with O_2_ to methanol, followed by the conversion of methanol and benzene to toluene, achieving 0.37% methane conversion and 80% selectivity to toluene at 330 °C and 11 bar.^[^ ^20^ ^]^ In our recent work, iron‐chabazite (Fe‐CHA) and iron‐aluminophosphate‐eighteen (Fe‐AEI) zeolites were identified as competitive bifunctional zeolites for the direct oxidation of methane to methanol and subsequent conversion of methanol to light olefins.^[^ ^17^, ^21^ ^]^ However, Fe‐zeolites were prone to over‐oxidation to CO_2_ at slightly elevated temperatures.^[^ ^17^, ^22^ ^]^

Zeolites, with their microporous structures and tunable acid sites, are renowned for their shape‐selective catalytic properties.^[^ ^23^ ^]^ While metal‐doped zeolites dominate methane conversion studies, their transition‐metal‐free counterparts utilize Brønsted or Lewis acid sites to activate small molecules, offering a sustainable and cost‐effective alternative. De Vos and co‐workers reported that transition‐metal‐free ultrastable USY zeolite can catalyze the formation of amides and amines using tert‐butylperoxy ethylhexyl carbonate as the oxidant.^[^ ^24^ ^]^ Chen and co‐workers noted that selective catalytic reduction of NO_x_ by methanol (CH_3_OH‐SCR) can be realized by using H‐FER zeolite without the participation of transition metals, due to the synergistic catalytic effect between Si─O(H)─Al sites and neighboring extra‐framework aluminum (EFAL) species.^[^ ^25^ ^]^ In our recent work, aluminosilicate FER zeolites were reported to efficiently and stably catalyze methane to methanol using N_2_O as the oxidant.^[^ ^26^ ^]^ Penta‐coordinated Al species in the extra‐framework were characterized as the possible active sites, and the two‐dimensional structure, combined with the preferential location of Al atoms at the short 8‐ring channels of FER, contributed to the efficient and stable conversion.^[^ ^26^, ^27^ ^]^

Acidic zeolites are versatile materials widely used in various reactions, such as catalytic cracking, isomerization, and MTO, due to their high surface area, thermal stability, and shape‐selective catalytic activity.^[^ ^1^, ^3^ ^]^ The strategic use of cascade catalytic systems could further enhance selectivity by spatially decoupling reaction steps, activating methane in one zeolite and sequentially assembling intermediates into olefins in another, thus mitigating over‐oxidation.^[^ ^20^, ^28^ ^]^ We have previously reported that methanol can be stably produced from methane over FER zeolite.^[^ ^26^ ^]^ Tandem conversion of methane to methanol on FER zeolite and methanol to light olefins on other acidic zeolites has not been specifically researched. The diversity of zeolite structures and acid properties presents a wealth of research possibilities.^[^ ^23^ ^]^

Herein, this study introduced a composite approach employing transition‐metal‐free zeolites for the upgrading of methane to light olefins via methanol as the intermediate. By orchestrating a sequence of zeolites with distinct structures and acidities, we aimed to control product distribution and formation rate, steering intermediates toward olefin production. The continuous flow design not only aligns with requirements for industrial scalability but also stabilizes reactive intermediates, enhancing efficiency. This method circumvented reliance on transition metals, addressing sustainability and cost challenges while capitalizing on the inherent thermal stability and shape selectivity of zeolite. Our findings underscore the potential of a cascade transition‐metal‐free zeolite catalytic system to revolutionize methane valorization, offering a greener paradigm for olefin synthesis.

## 2 Results and Discussion

### 2.1 Catalytic Performance of FER/Acidic Zeolites

**Figure 1:**
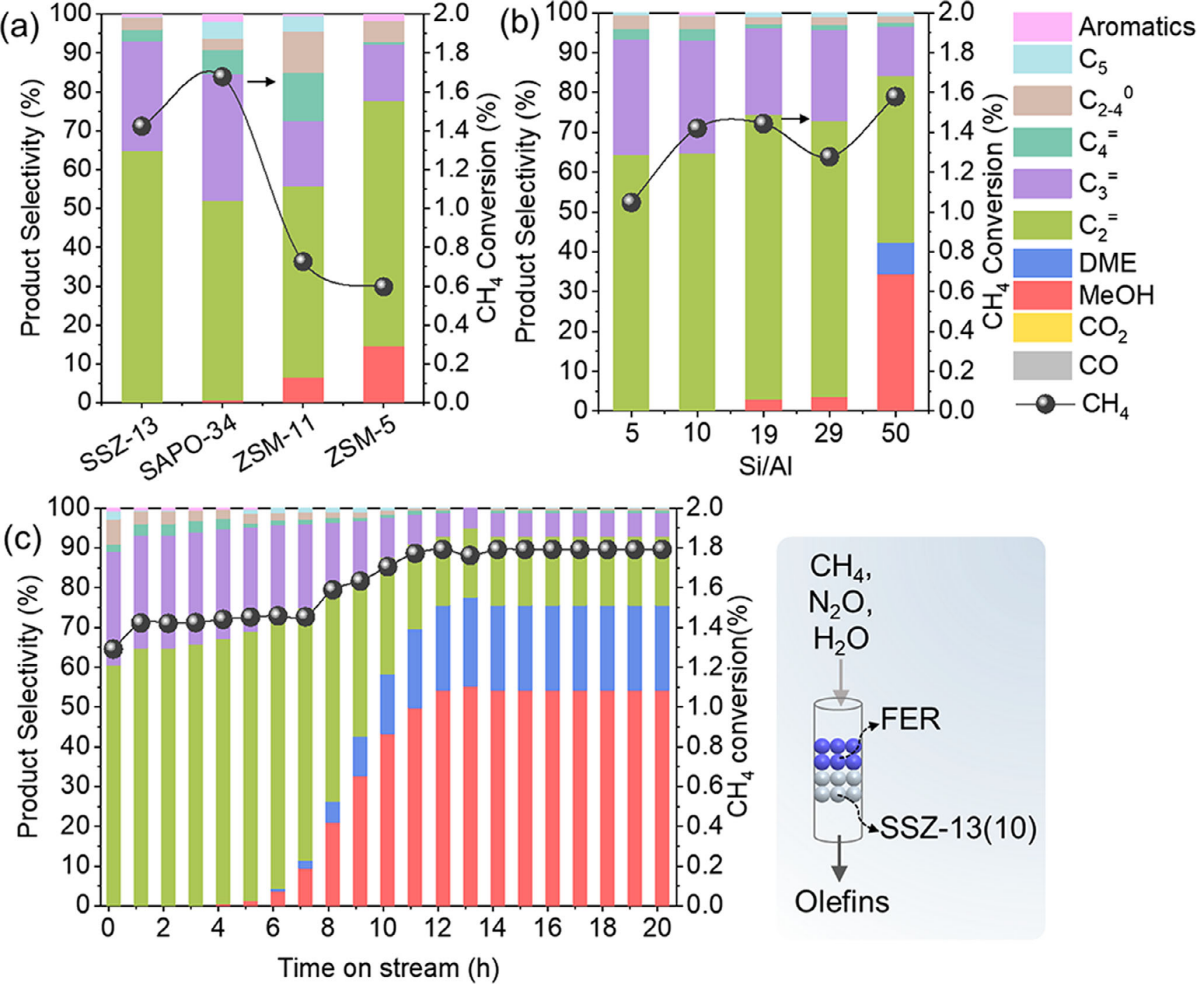
Catalytic performance in the direct methane oxidation. a) CH_4_ conversion and product distribution over composite FER and acidic zeolites with different topological structures. b) Effect of Si/Al ratio in SSZ‐13 on catalytic performance in FER/SSZ‐13 systems. Reaction conditions: 350 °C, 50 mg FER + 50 mg acidic zeolite (dual‐bed), CH_4_/N_2_O/H_2_O/Ar = 10/10/2/3 ml·min^−1^, WHSV = 15 000 ml·g^−1^·h^−1^, TOS = 0.17 h. c) Stability test of FER/SSZ‐13(10) (50 mg + 50 mg) in dual‐bed mode under identical conditions.

As depicted in **Figure** **1**a, methane was directly converted to hydrocarbons when FER was followed by acidic zeolites, with the product distribution depending on the framework topology of the following acidic zeolite (Figures S1–S7 and Tables S1–S3, Supporting Information). While all acidic zeolites selectively produced light olefins, the medium‐pore zeolites ZSM‐11 and ZSM‐5 did not fully convert the methanol intermediate to hydrocarbons. In contrast, the small‐pore zeolites SSZ‐13 and SAPO‐34 almost completely converted methanol to hydrocarbons, resulting in a higher hydrocarbon formation rate than the medium‐pore zeolites (Figure S7, Supporting Information). Methanol was detected as an unreacted intermediate in products over ZSM‐11 and ZSM‐5, due to the insufficient acid strength and low acid site density in these zeolites, which prevented complete methanol conversion in the tandem catalytic system. (Figures  S4 and S6, Table S3, Supporting Information). In addition, the product distributions of FER/ZSM‐11 and FER/ZSM‐5 differed from those in the traditional MTO reaction due to the insufficient methanol and external mass transfer limitations.^[^ ^29^, ^30^, ^31^, ^32^ ^]^ When the transition‐metal‐free acidic zeolites were tested independently in the direct methane oxidation reaction, weak activity was obtained (Figure  S5, Supporting Information). Catalytic performance of SAPO‐34 and ZSM‐5 in MTO conversion was evaluated (Figure S8, Supporting Information), achieving ≈80% and 16% ethylene‐butene (C_2_^=^ ‐C_4_^=^) olefin selectivity in the initial stage, respectively. This was slightly lower than the tandem catalysis of methane to methanol and methanol to olefins reaction (Figure 1a). Additionally, MTO performance in a dual‐bed FER and acidic zeolites mode was explored (Figure S9, Supporting Information). At 350 °C, C_2_^=^ ‐C_4_^=^ olefin selectivity dropped below 50%, attributed to the presence of FER and the high acid site density of acidic zeolites.^[^ ^26^, ^27^ ^]^ Thus, the generated hydrocarbon over composite FER and acidic zeolites was confirmed to be produced by methane oxidation to methanol over FER zeolite, followed by methanol being converted to hydrocarbon over acidic zeolites. These results indicate that small‐pore zeolites are more suitable than medium‐pore zeolites for the conversion of methane to olefins via methanol as the intermediates, being cascaded with FER zeolite.

Figure 1b displays the catalytic performance of sequenced FER and SSZ‐13 zeolites of varying Si/Al ratios in the methane oxidation reaction (Figures S10–S16 and Tables S1–S3, Supporting Information). Hydrocarbon selectivity decreased from 100% to 57.6% as the Si/Al ratio increased from 5 to 50. This decline indicated that insufficient acid site density cannot effectively catalyze the conversion of the methanol intermediate to hydrocarbons. This was further supported by the concomitant increase in the selectivity and formation rate of methanol and dimethyl ether (DME) (Figures S15 and S16 and Table S3, Supporting Information). Notably, SSZ‐13 zeolites alone exhibited negligible activity in direct methane oxidation (Figure S17, Supporting Information), confirming that their role was confined to methanol conversion in the cascade catalytic systems. Only SSZ‐13 zeolites with sufficient acid site density and strength, namely SSZ‐13(5) and SSZ‐13(10), produced hydrocarbons as the main products (Figure S13, Supporting Information). However, SSZ‐13(5), despite having higher acid site density and stronger acid strength, exhibited lower methane conversion and lower hydrocarbon formation rate than SSZ‐13(10) when coupled with FER (Table  S2, Supporting Information). This counterintuitive result was likely due to the extra‐framework aluminum (EFAl) species in SSZ‐13(5) (Figure S12, Supporting Information), especially the hexacoordinated Al (EFAl_VI_), leading to pore blockage, as evidenced by its lower total pore volume compared to SSZ‐13(10) (Figure  S14 and Table S1, Supporting Information). In the FER/SSZ‐13 cascade catalytic system, C_2_^=^ was consistently the dominant hydrocarbon product. This product distribution was similar to that observed in the MTO reaction catalyzed by SSZ‐13 zeolites with different Si/Al ratios.^[^ ^33^ ^]^ These results suggested that the hydrocarbon pool mechanism, specifically the aromatic‐based cycle, governed the tandem conversion of methane‐derived methanol to olefins over the acidic SSZ‐13 component.

Furthermore, the FER/SSZ‐13(10) tandem catalysis demonstrated stable conversion of methane to olefins for 7 h, maintaining 100% hydrocarbon selectivity and 1.4% methane conversion, as depicted in Figure 1c. Notably, this 1.4% methane conversion was primarily attributable to the FER zeolite, consistent with our previous work using only 50 mg of FER zeolite.^[^ ^26^ ^]^ The presence of the acidic zeolite component did not enhance methane conversion; conversely, it introduced additional mass transfer resistance for reactants. The phenomenon differed from that observed in the single‐crystal zeolite with bifunctional sites reported in our latest work,^[^ ^27^ ^]^ in which the MTO reaction promotes the first step of methane conversion. This difference likely arises because the single‐crystal zeolite exhibits both a short propagation distance between bifunctional sites and lower mass transfer resistance compared to the composite zeolites. In the case of medium‐pore zeolites like ZSM‐11 and ZSM‐5, both methane and methanol conversion were obstructed, as evidenced by the lower methane conversion and unreacted methanol observed in Figure 1a. Deactivation during the stability test was attributed to carbon deposition on the SSZ‐13(10) component. While the FER component remained active and stable for methane oxidation to methanol, achieving 1.8% methane conversion with the (MeOH + 2×DME) formation rate of 80 µmol·g^−1^·min^−1^ and selectivity of 75%.

### 2.2 Catalytic Performance of the FER/SAPO‐34 Tandem Catalysis System

#### 2.2.1 Mass Ratios of FER/SAPO‐34

**Figure 2:**
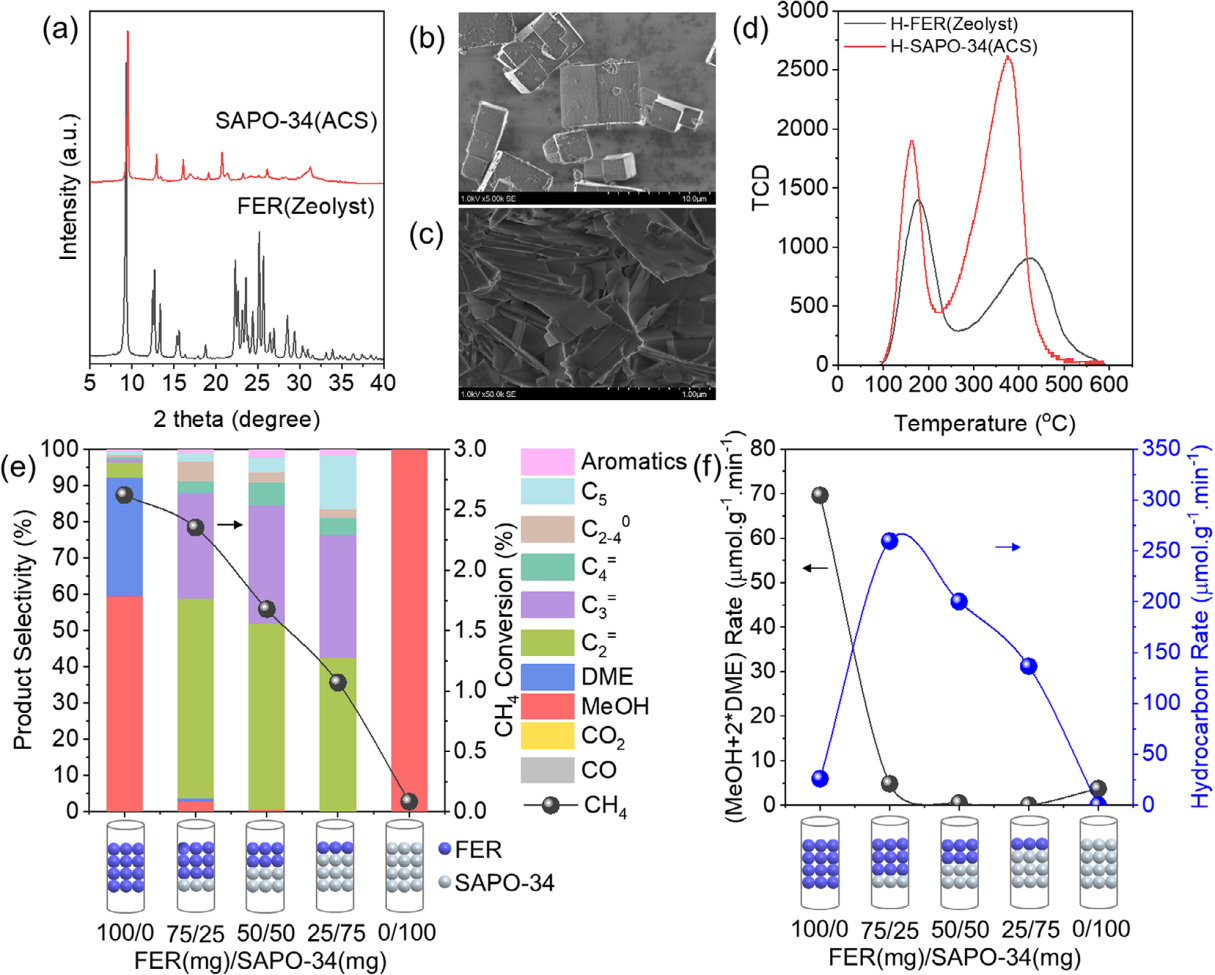
a) XRD patterns of FER and SAPO‐34 zeolites. SEM image of b) SAPO‐34 and c) FER zeolite. d) Compare NH_3_‐TPD profiles of FER and SAPO‐34 zeolites. e) Methane conversion and product distribution, and f) product formation rates over a tandem catalyst with varying FER/SAPO‐34 mass ratios. Reaction conditions: 350 °C, FER (*x* mg) + SAPO‐34 (100‐*x* mg), CH_4_/N_2_O/H_2_O/Ar = 10/10/2/3 mL·min^−1^, WHSV = 15 000 ml·g^−1^·h^−1^, TOS = 0.17 h.

SAPO‐34 was selected for reaction optimization based on its superior hydrocarbon yield when coupled with FER (Figure 1a), excellent reproducibility as a commercial zeolite (**Figure** **2**a–d), and negligible activity in direct methane oxidation at 350 °C (Figure S5, Supporting Information). Figure 2e illustrates methane conversion and product distribution as functions of the FER/SAPO‐34 mass ratio in a dual‐bed configuration. Using 100 mg FER alone yielded methanol as the primary product with 2.6% CH_4_ conversion, consistent with its established capability for selective oxidation of methane to methanol over penta‐coordinated Al species in its two‐dimensional 8/10‐ring topology.^[^ ^26^ ^]^ SAPO‐34 alone exhibited merely 0.08% CH_4_ conversion, attributed to the trace formation of active Al species. Comparative ^2^⁷Al MAS NMR of fresh and spent SAPO‐34 (Figure  S18, Supporting Information) revealed only marginal changes in penta‐coordinated Al intensity, confirming insufficient active site generation. As the FER mass decreased and the SAPO‐34 mass increased proportionally (total catalyst = 100 mg), hydrocarbon products gradually dominated. CH_4_ conversion decreased from 2.4% to 1.2% (Figure S19, Supporting Information). Product distribution depended critically on methanol output determined by the mass of FER zeolite, and acid site availability determined by the mass of SAPO‐34 zeolite. The optimal mass ratio of 50/50 (FER/SAPO‐34) achieved the peak hydrocarbon formation rate of 282 µmol·g^−1^·min^−1^ and hydrocarbon selectivity of 96% (Figure 2f). Notably, when FER was fixed at 25 mg, varying SAPO‐34 mass (25–200 mg) minimally affected conversion or distribution (Figure S20, Supporting Information), demonstrating that methanol production (FER‐dependent), not acid site quantity (SAPO‐34‐dependent), was the rate‐limiting factor. Stability testing of 25 mg FER + 100 mg SAPO‐34 at 350 °C confirmed sustained production of light olefins (20 h), with consistent selectivity to ethylene (C_2_⁼, ≈60%) and propylene (C_3_⁼, ≈30%).

### 2.3 Reaction Route

**Figure 3:**
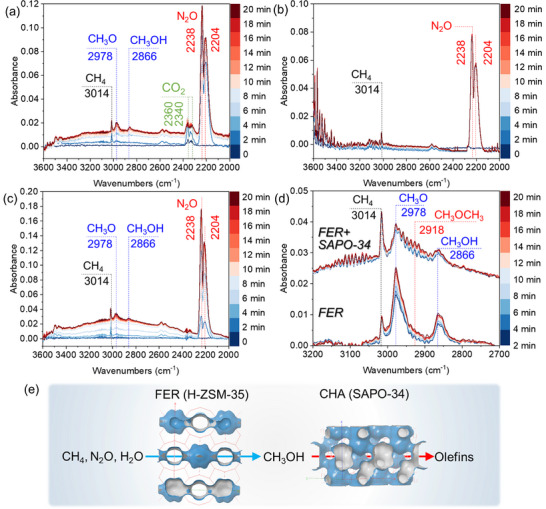
In situ DRIFTS spectra during co‐feeding of CH_4_ (5 mL min^−1^), N_2_O (5 mL min^−1^) and Ar (500 mL min^−1^): a) FER at 350 °C (0–20 min), b) SAPO‐34 at 350 °C (0–20 min), c) FER+SAPO‐34 physical mixture at 350 °C (0–20 min). d) Comparative spectra of FER and FER+SAPO‐34 at 400 °C (0–20 min). e) Proposed reaction pathway: methane oxidation to methanol on FER zeolite, followed by methanol conversion to olefins on SAPO‐34 zeolite.

In situ diffuse reflectance infrared Fourier transform spectroscopy (DRIFTS) of methane oxidation over the transition‐metal‐free zeolites was performed to elucidate the methane oxidation mechanism. **Figure** **3**a revealed progressive intensification of the CH_3_O species peak at 2978 cm^−1^ on FER zeolite at 350 °C during co‐feeding of CH_4_/N_2_O/Ar (0–20 min), followed by emergence of a weak CH_3_OH peak at 2866 cm^−1^.^[^ ^13^, ^34^, ^35^ ^]^ These observations confirmed the mechanism involving methoxy intermediates and support for methane‐to‐methanol oxidation.^[^ ^13^, ^34^ ^]^ In contrast, SAPO‐34 exhibited no detectable CH_3_O or CH_3_OH signals under identical conditions (Figure 3b), consistent with its negligible direct methane oxidation activity. When the physically mixed FER and SAPO‐34 catalyst was tested, both CH_3_O (2978 cm^−1^) and CH_3_OH (2866 cm^−1^) peaks appeared (Figure 3c). The broadening CH_3_O peak over time suggested the formation of additional species. Comparative spectra at 400 °C (Figure 3d) showed distinct band broadening on FER+SAPO‐34, with a new peak at 2918 cm^−1^ attributable to CH_3_OCH_3_.^[^ ^34^ ^]^ This observation is consistent with our recent findings on a single bifunctional CHA zeolite catalyst.^[^ ^36^ ^]^ Notably, light olefins were undetected due to limited mass transfer between active sites in the DRIFTS configuration. These facts suggest that the tandem pathway (Figure 3e) which CH_4_ is oxidized to C_1_ intermediates on FER, and then the intermediates migrate to SAPO‐34 acid sites, and finally, C─C coupling forms light olefins.^[^ ^37^ ^]^

### 2.4 Spatial Proximity Effects

**Figure 4:**
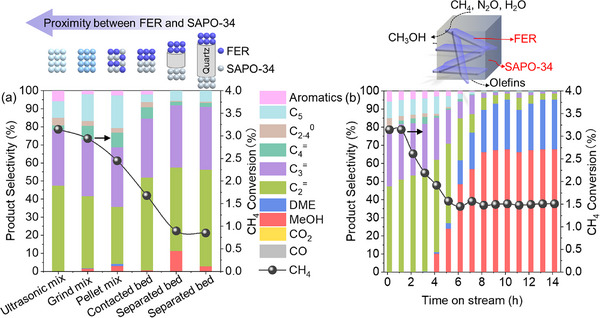
a) Effect of catalyst configuration on catalytic performance at 350 °C. b) Stability test of ultrasonically mixed FER/SAPO‐34 (50 mg + 50 mg). Reaction conditions: CH_4_/N_2_O/H_2_O/Ar = 10/10/2/3 mL·min^−1^, WHSV = 15 000 ml·g^−1^·h^−1^.

Catalytic performance strongly depended on the spatial distance between FER and SAPO‐34 components, which was analogous to metal‐acid site proximity effects in bifunctional zeolites.^[^ ^29^ ^]^ We engineered increasing separation via ultrasonic mixing (closest), grind mixing, pellet mixing, direct dual‐bed contact, and dual‐bed separated by quartz wool (farthest). **Figure** **4**a demonstrates that increased separation reduced the methane conversion from 3.1 to 0.85% and the hydrocarbon formation rate from 411 to 95 µmol·g^−1^·min^−1^. This proximity dependence aligned with bifunctional catalysis principles.^[^ ^38^, ^39^, ^40^ ^]^ Crucially, closer spatial integration maximized olefin production,^[^ ^29^ ^]^ mirroring recent findings in propane dehydrogenation.^[^ ^41^ ^]^ Separation by 1 or 2 cm of quartz wool showed negligible differences. The stability test using ultrasonically mixed FER/SAPO‐34 (50:50 mg) demonstrated 100% hydrocarbon selectivity for 4 h (Figure 4b). Subsequent decline to 95% oxygenate (MeOH + DME) selectivity and 85 µmol·g^−1^·min^−1^ formation rate (MeOH + 2×DME) after 10 h (Figure 4b; Figure S21, Supporting Information) indicated the deactivation of SAPO‐34 zeolite, halting methanol‐to‐olefins conversion.

### 2.5 Thermodynamics and Kinetics

**Figure 5:**
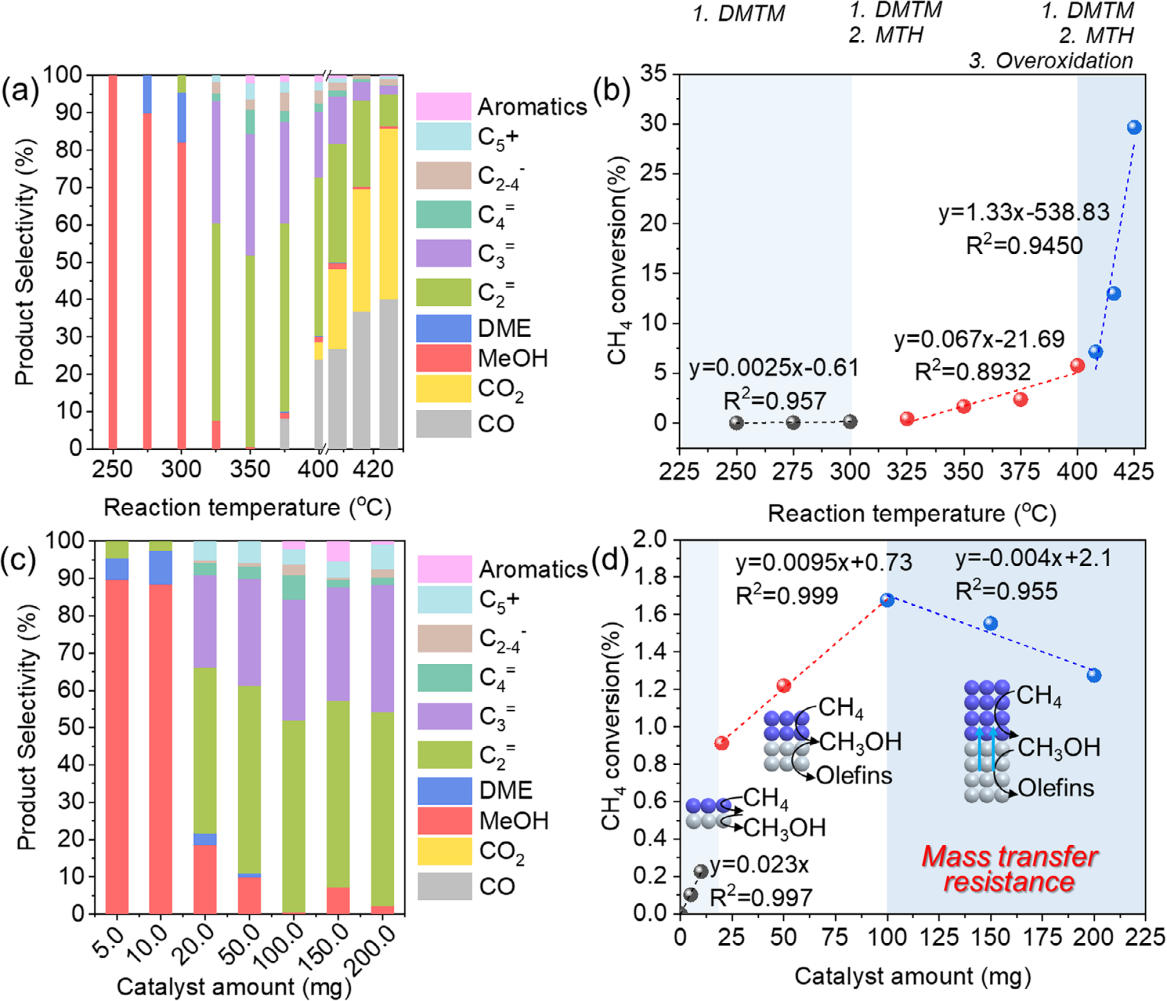
a) Temperature‐dependent product distribution (250–425 °C), b) correlation between temperature and methane conversion. Reaction conditions: 50 mg FER + 50 mg SAPO‐34 (dual‐bed), CH_4_/N_2_O/H_2_O/Ar = 10/10/2/3 mL·min^−1^, WHSV = 15 000 ml·g^−1^·h^−1^, TOS = 0.17 h. c) Catalyst loading‐dependent product distribution, d) correlation between total catalyst mass (5–200 mg) and methane conversion. Reaction conditions: 350 °C, FER (2.5–100 mg) + SAPO‐34 (2.5–100 mg) (dual‐bed), TOS = 0.17 h.

Reaction temperature critically regulates activity and product distribution (Figure S22, Supporting Information). Tandem conversion over FER/SAPO‐34 (50:50 mg) exhibits distinct regimes (**Figure** **5**a). Dominant oxygenates (MeOH/DME) formed at 250–300 °C; hydrocarbons prevailed as primary products at 325–400 °C; and CO/CO_2_ dominated via over‐oxidation at temperatures higher than 425 °C. This profile is consistent with transition‐metal‐containing zeolites (e.g., Fe/Cu‐zeolites),^[^ ^17^, ^18^, ^34^ ^]^ confirming the inherent exothermicity of methane‐to‐methanol conversion, which is thermodynamically favored at lower temperatures but kinetically limited by C─H bond activation. Figure 5b quantifies temperature‐dependent methane conversion. In stage I (250–300 °C), methane conversion slowly increased (slope = 0.0025% °C^−1^), governed by kinetically direct methane‐to‐methanol oxidation. In stage II (300–400 °C), methane conversion was accelerated (slope = 0.067% °C^−1^) driven by a tandem catalyst of methane to methanol, followed by methanol to hydrocarbons. In stage III (>400 °C), methane conversion rapidly surged (slope = 1.33% °C^−1^) due to the combination of DMTM (direct methane to methanol), MTH (methanol to hydrocarbons), and over‐oxidation to CO and CO_2_. Thermodynamic analysis revealed a sequential pathway: partial oxidation of CH_4_ to CH_3_OH occurred on FER zeolite, acid‐catalyzed conversion of CH_3_OH to hydrocarbons proceeded on SAPO‐34, and concurrent over‐oxidation to CO_2_ occurred at elevated temperatures. In this tandem catalyst, methanol serves as the essential intermediate before the C─C bond formation via acid catalysis, with subsequent oxidation representing an undesired side reaction.

The catalytic performance of the FER/SAPO‐34 cascade catalytic system (1:1 mass ratio) exhibited significant dependence on total catalyst loading, or the length of the catalyst bed. As demonstrated in Figure 5c, methanol was the main product when the total catalyst was less than 10 mg. Hydrocarbons become primary products by gradually increasing the catalyst mass. The lower catalyst mass resulted in higher methanol selectivity, whereas the higher catalyst mass led to lower CH_4_ conversion. This indicates that the reaction rate of converting methane to methanol is significantly faster than that for converting methanol to olefins. These findings differ from our latest work using a single CHA zeolite as a bifunctional catalyst for converting methane to olefins via methanol as the intermediate,^[^ ^36^ ^]^ suggesting the significant external mass transfer limitations in the composite catalyst system. Figure 5d reveals three distinct regimes for methane conversion versus catalyst mass. In stage I, CH_4_ conversion rapidly increased (slope = 0.023%·mg^−1^) with minimal mass transfer resistance along with the catalyst mass. In stage II, reduced conversion growth was obtained (slope = 0.0095%·mg^−1^) with emerging mass transfer resistance. In stage III, conversion declined (slope = −0.004%·mg^−1^) with severe diffusion constraints in the extended catalyst bed. The inverse relationship between conversion and catalyst mass beyond 100 mg demonstrated that excessive bed length impeded reactant transport. A kinetic study of a representative combination of FER/SAPO‐34 zeolite catalyst indicated a sequential reaction pathway in which CH_4_ was partially oxidized to CH_3_OH before secondary downstream of acidic catalyzing CH_3_OH to hydrocarbon without the over‐oxidation reaction to CO_2_ by controlling the reaction temperature and catalyst amount.

### 2.6 H_2_O Partial Pressure

**Figure 6:**
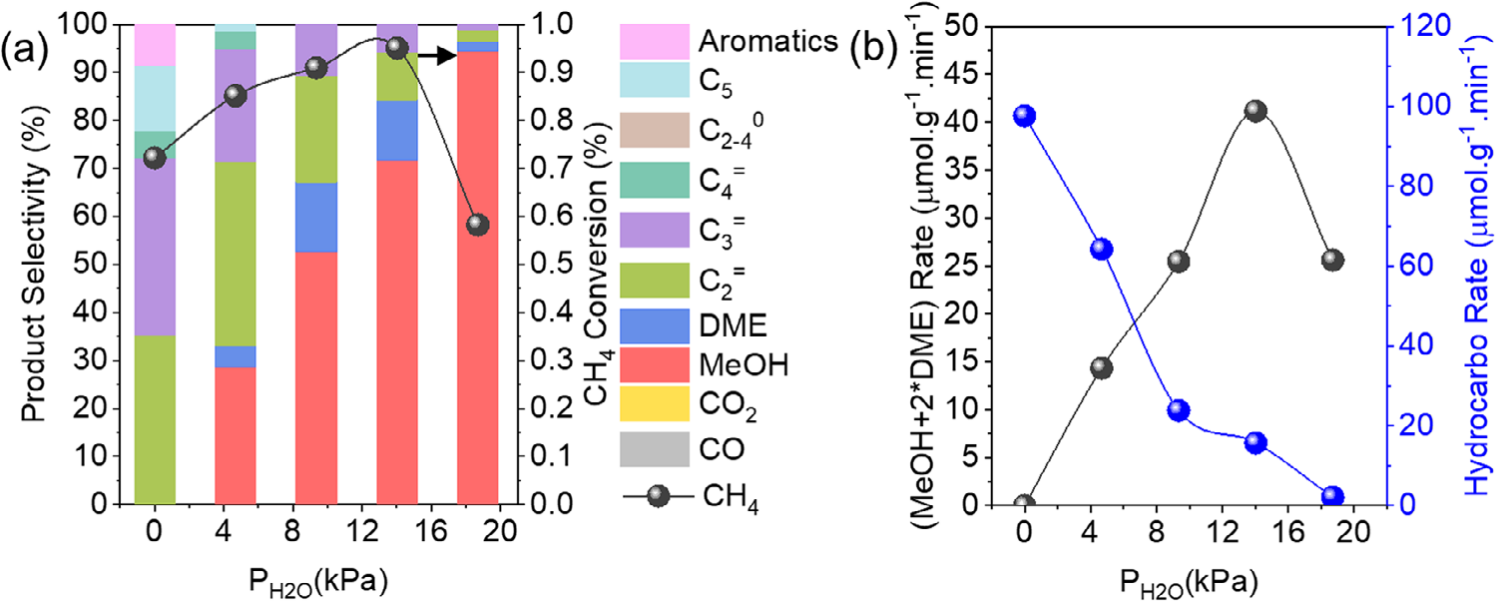
Influence of water partial pressure on a) product distribution and methane conversion, and b) product formation rates. Reaction conditions: 350 °C, 50 mg FER + 50 mg SAPO‐34 (dual‐bed), CH_4_/N_2_O/H_2_O/Ar = 10/10/x/(45‐*x*) mL·min^−1^ (*x* = 0–12), WHSV = 39 000 ml·g^−1^·h^−1^, TOS = 0.17 h.

The partial pressure of H_2_O (*P_H2O_*) critically governs reaction pathways in cascade transition‐metal‐free zeolite catalytic systems, modulating methane activation kinetics, methanol stability, and olefin selectivity.^[^ ^42^, ^43^, ^44^ ^]^ By adjusting *P_H2O_*, the balance between methane activation, methanol protection, and olefin formation can be finely tuned. As shown in **Figure** **6**, the CH_4_ conversion initially increased from 0.72% to 0.95% with *P_H2O_* increasing from 0 to 14 kPa, and then decreased to 0.58% by further increasing *P_H2O_* from 14 to 19 kPa. Accordingly, the selectivity and formation rate of hydrocarbons declined with increasing *P_H2O_*, and conversely, the methanol selectivity progressed. This inverse relationship revealed the dual role of water. On the one hand, water promoted methane‐to‐methanol oxidation by serving as a reactant in N_2_O activation^[^ ^43^ ^]^ and facilitating methanol desorption via H‐bonding as a solvent.^[^ ^44^, ^45^ ^]^ On the other hand, water inhibited methanol‐to‐olefins conversion by competing with methanol for acid sites, inducing structural hydrolysis at elevated concentrations,^[46]^ and suppressing hydrocarbon pool formation. Consequently, in the integrated process, H_2_O from upstream DMTM reactions must be carefully regulated to avoid cascading impacts on the downstream MTO reaction.

## 3 Conclusion

In conclusion, this work demonstrated a transformative approach for continuous conversion of methane to olefins through cascade transition‐metal‐free FER, acidic zeolites. By leveraging the efficient, stable production of methanol from methane over FER zeolite, the diversity of acidic zeolites, adjustable acid amount, spatial decoupling of reaction steps, this work circumvented longstanding challenges associated with the inertness of methane, over‐oxidation, and realized direct conversion of CH_4_, N_2_O to light olefins via methanol as the intermediates with adjustable product distribution. The system achieved peak performance at 90% selectivity of light olefins with 3.1% CH_4_ conversion, minimal CO_2_ formation. This outperforms conventional transition‐metal catalysts plagued by deactivation, environmental concerns. Crucially, the strategy not only eliminated reliance on transition metals but also aligned with global sustainability goals by upgrading methane into high‐value chemicals, providing a blueprint for earth‐abundant catalytic systems. Our findings unveiled the untapped potential of the cascade zeolite catalytic system in C1 chemistry, establishing a green route for olefin synthesis from natural gas.

## Conflict of Interest

The authors declare no conflict of interest.

## Supporting information



Supporting Information

## Data Availability

The data that support the findings of this study are available from the corresponding author upon reasonable request.
