# Supplementary material for: Transition‐Metal‐Free Zeolite Composites for Tandem Catalytic Conversion of Methane to Light Olefins
Source: Adv Sci (Weinh). 2025 Oct 13;13(2):e15145. doi: 10.1002/advs.202515145 (PMC12786352; doi:10.1002/advs.202515145)
Supplement: Supplementary file 1 — Supporting Information [file ADVS-13-e15145-s001.docx]

**Supporting information**

**Transition-metal-free zeolite composites for tandem catalytic conversion of methane to light olefins**

Peipei Xiao,^1^ Hiroto Toyoda,^1^ Yuqin Sun,^1^ Yong Wang,^1^ Herman. Gies,^1,2^ Toshiyuki Yokoi*^1,3^

^1^ Institute of Integrated Research, Institute of Science Tokyo, 4259 Nagatsuta, Midori-ku, Yokohama 226-8501, Japan

^2^ Institute of Geology, Mineralogy and Geophysics, Ruhr-University Bochum, Bochum 44780, Germany

^3^ iPEACE223 Inc., Konwa Building, 1-12-22 Tsukiji, Chuo-ku, Tokyo, 104-0045, Japan

E-mail: [yokoi@cat.res.titech.ac.jp](mailto:yokoi@cat.res.titech.ac.jp)

**Catalyst preparation**

FER and SAPO-34 zeolites were commercially obtained from Zeolyst (CP914C, NH₄⁺-form) and ACS MATERIAL (MSSA3421, Type B), respectively. All samples were calcined at 550 °C for 5 h in air before use.

SSZ-13 zeolites with target Si/Al ratios (x = 5, 10, 25, 50, 100) were hydrothermally synthesized using N,N,N-trimethyl-1-adamantylammonium hydroxide (TMAdaOH) as organic structure-directing agent (OSDA), following our established protocol.^[1]^ Briefly, the alkaline solution containing TMAdaOH, NaOH, and Al(OH)_3_ was mixed with fumed silica (Cab-O-Sil M5) and 5wt.% seed (SSZ-13 zeolite). The prepared gels with the molar ratio of 1 SiO_2_: 1/2*x* Al_2_O_3_: 0.2 NaOH: 0.2 TMAdaOH: 30 H_2_O with 5 wt.% seed (SSZ-13) (*x*=5, 10, 25, 50, 100) were crystallized at 150 °C in a rotating oven for 5 days. After washing, filtering, and drying, the obtained samples were named as-SSZ-13-*x*, where *x* meant the Si/Al ratio in the synthesis gel. Subsequently, the as-synthesized products were calcined at 550 °C for 10 h in air to remove OSDA. Afterward, the calcined samples were exchanged twice with 2.5 M NH_4_NO_3_ aqueous solution at 80 °C for 3 h to get the NH_4_-form samples. Finally, the NH_4_-type samples were calcined at 550 ^o^C for 5 h in air to get H-type SSZ-13(*x*) zeolites.

ZSM-11 zeolite was synthesized based on the literature using TBAOH as the OSDA.^[2]^ Specifically, the alkaline solution containing TBAOH, NaOH, and Al(NO_3_)_3_ was stirred fully with TEOS. The prepared gel with the molar ratio of 1 SiO_2_: 0.025Al_2_O_3_: 0.08 NaOH: 0.2 TBA: 10H_2_O was crystallized at 170 °C in a rotating oven for 3 days. After washing, filtering, and drying, the as-synthesized product was calcined at 550 °C for 10 h in air to remove OSDA. Afterward, the calcined sample was exchanged twice with 2.5 M NH_4_NO_3_ aqueous solution at 80 °C for 3 h to get the NH_4_-form sample. Finally, the NH_4_-type sample was calcined at 550 ^o^C for 5 h in air to get H-type ZSM-11 zeolite.

ZSM-5 zeolite was synthesized according to our previous work in the presence of TPAOH and sodium.^[3]^ Specifically, the alkaline solution containing TPAOH, NaOH, and Al(NO_3_)_3_ was stirred fully with TEOS. The prepared gel with the molar ratio of 1 SiO_2_: 0.02Al_2_O_3_: 0.1 NaOH: 0.25 TPA: 30 H_2_O was crystallized at 170 °C in a rotating oven for 3 days. After washing, filtering, and drying, the as-synthesized product was calcined at 550 °C for 10 h in air to remove OSDA. Afterward, the calcined sample was exchanged twice with 2.5 M NH_4_NO_3_ aqueous solution at 80 °C for 3 h to get the NH_4_-form sample. Finally, the NH_4_-type sample was calcined at 550 ^o^C for 5 h in air to get H-type ZSM-5 zeolite.

**Catalyst characterization**

XRD patterns were collected on a Rint-Ultima III (Rigaku) using a Cu Kα X-ray source (40 kV, 40 mA).

Elemental analyses of samples were performed on an inductively coupled plasma-atomic emission spectrometer (ICP-AES, Shimadzu ICPE-9000).

Field-emission scanning electron microscopic (FE-SEM) images of the powder samples were obtained on SU9000 (Hitachi) microscope operating at 1 kV.

Solid-state ^27^Al MAS NMR spectra were measured on a JEOL ECA-600 spectrometer at a resonance frequency of 156.4 MHz using a 4 mm sample rotor with a spinning rate of 15.0 kHz. The ^27^Al chemical shift was referenced to -0.54 ppm of AlNH_4_(SO_4_)_2_·12H_2_O.

Temperature-programmed ammonia desorption (NH_3_-TPD) profiles were recorded on Multitrack TPD equipment (Japan BEL). Typically, 25 mg of catalyst was pretreated at 600 ^o^C in He (50 mL min^−1^) for 1 h and then cooled to 100 ^o^C. Before the adsorption of NH_3_, the sample was evacuated at 100 ^o^C for 1 h. Approximately 2500 Pa of NH_3_ was allowed to contact with the sample at 100 ^o^C for 10 min. Subsequently, the sample was evacuated to remove weakly adsorbed NH_3_ at the same temperature for 30 min. Finally, the sample was cooled to 100 ^o^C and heated from 100 to 600 ^o^C at a ramping rate of 10 ^o^C min^−1^ in a He flow (50 mL min^−1^). A thermal conductivity detector (TCD) was used to monitor desorbed NH_3_. The amount of acid sites was determined by the fitting peak area of the profiles.

Nitrogen adsorption and desorption measurements to obtain information on the micro- and meso-porosities were conducted at -196 ^o^C on a Belsorp-mini II (MicrotracBEL).

The *in situ* diffuse reflectance infrared Fourier transform spectroscopy (DRIFTS) experiment was performed on a JASCO FTIR-4600 with an MCT detector. First, the catalyst was treated under Ar flow (500 mL/min) at 500 °C for 1 h, and then the catalyst was cooled down to 350 °C. The background spectrum was obtained at 350 ^o^C in Ar flow. Then, the catalyst sample was exposed to a CH_4_/N_2_O/Ar mixture (5 mL/min CH_4_, 5 mL/min N_2_O, 500 mL/min Ar) at 350 and 400 ^o^C, and the spectrum was recorded at 0-20 min.

**Catalytic tests in methane oxidation reaction**

Continuous methane oxidation was conducted in a fixed-bed flow reactor. Typically, 100 mg of catalyst pellets (500–1000 μm) were loaded into a quartz tube reactor (ID 4 mm) housed in an electric furnace. After pretreatment in flowing air at 500 °C for 1 h, reactions were performed at 350 °C. Effluent gases were analyzed using two online GC systems (Shimadzu GC-2014):

- GC-1: Equipped with a Shincarbon ST 50/80 packed column (3 mm × 6 m) and TCD with methanizer, quantifying H₂, N₂O, CO, CO₂, and CH₄.
- GC-2: Equipped with an HP-PLOT Q capillary column (0.53 mm × 30 m × 40 μm) and FID, quantifying CH₄, methanol (MeOH), dimethyl ether (DME), olefins/alkanes.

Carbon-based metrics were calculated as follows:

Methane Conversion (*X*_CH4_):

*X*_CH4_ =$\frac{\sum\left( ni\cdot Ci \right)}{\sum\left( ni\cdot Ci \right)+nCH4}\times100\%$

where *ni*= carbon number in product *i*, *Ci* = molar flow rate of i*i* (μmol·min⁻¹),*n_CH4_* = unconverted CH₄ flow rate.

N₂O Conversion (*X*_N2O_​):

*X*_N2O_ =$\frac{nN2O,in-nN2O, out}{nN2O,in}\times100\%$

Product Selectivity (*Si*​):

*S*_i_ =$\frac{ni*Ci}{\sum(nj*Cj)}\times100\%$

Product Yield (*Yi​*):

*Y*_i_ =$\frac{ni*Ci}{\sum(nj*Cj)+nCH4}\times100\%=$*X*_CH4_*​​⋅S*_i_

Formation Rate (*ri*​):

*r*_i_ =$\frac{Yi *FCH4}{mcat}$

where *F_CH4_, in​*= inlet CH₄ flow rate (μmol·min⁻¹), *m_cat​_*= catalyst mass (g), *r_hydrocarbons_*= 2*(*r_C2_^=^ + r_C2_^-^*) + 3*(*r_C3_^=^ + r_C3_^-^*) + 4*(*r_C4_^=^ + r_C4_^-^*) + 5*(*r_C5_^=^ + r_C5_^-^*).

**Catalytic test in methanol to olefins (MTO) reaction**

The MTO reaction was performed using a fixed-bed reactor connected to an online gas chromatograph (GC-2014, Shimadzu) equipped with an HP-PLOT/Q capillary column and a flame ionization detector. The 50/80 mesh zeolite pellets without a binder were placed in a 6 mm quartz tubular flow reactor. The pretreatment was conducted at 500 °C for 30 min under Ar (20 mL·min^−1^). After the pretreatment, the reactor was cooled to 350 °C, and the MTO reaction commenced. The pressure of methanol was set at 10 kPa with Ar gas as the carrier; the weight-to-feed ratio (W/F) for methanol was set at 34 g·h·mol^−1^. The product stream was analyzed using a system that automatically injected the product into a gas chromatograph connected directly to the outlet of the reactor via a heated transfer line.

The conversion and selectivity were calculated as follows:

𝐶𝑜𝑛𝑣𝑒𝑟𝑠𝑖𝑜𝑛 𝑜𝑓 𝑚𝑒𝑡ℎ𝑎𝑛𝑜𝑙 [%] = 1 ‒ × 100


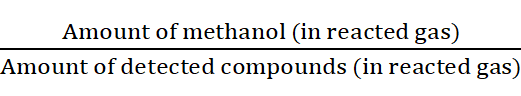

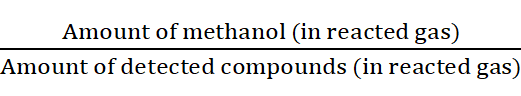


𝑃𝑟𝑜𝑑𝑢𝑐𝑡 𝑠𝑒𝑙𝑒𝑐𝑡𝑖𝑣𝑖𝑡𝑦 [%] = × 100


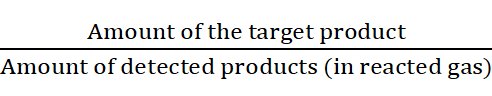

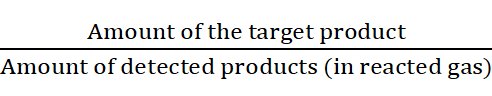


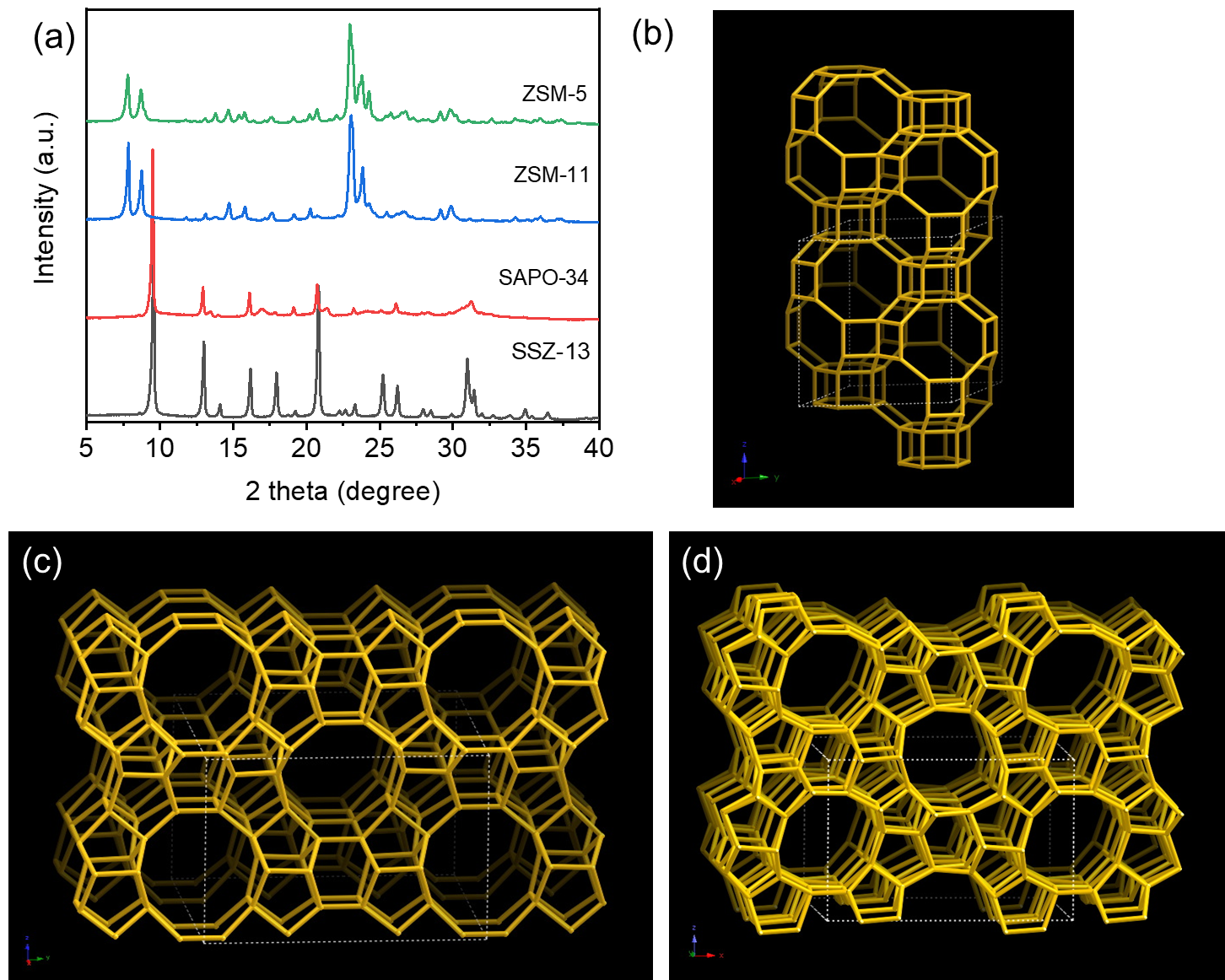


**Figure S1** (a) XRD patterns of zeolites. Topological structures of (b) CHA (SSZ-13, SAPO-34), (c) MEL (ZSM-11), (d) MFI (ZSM-5).


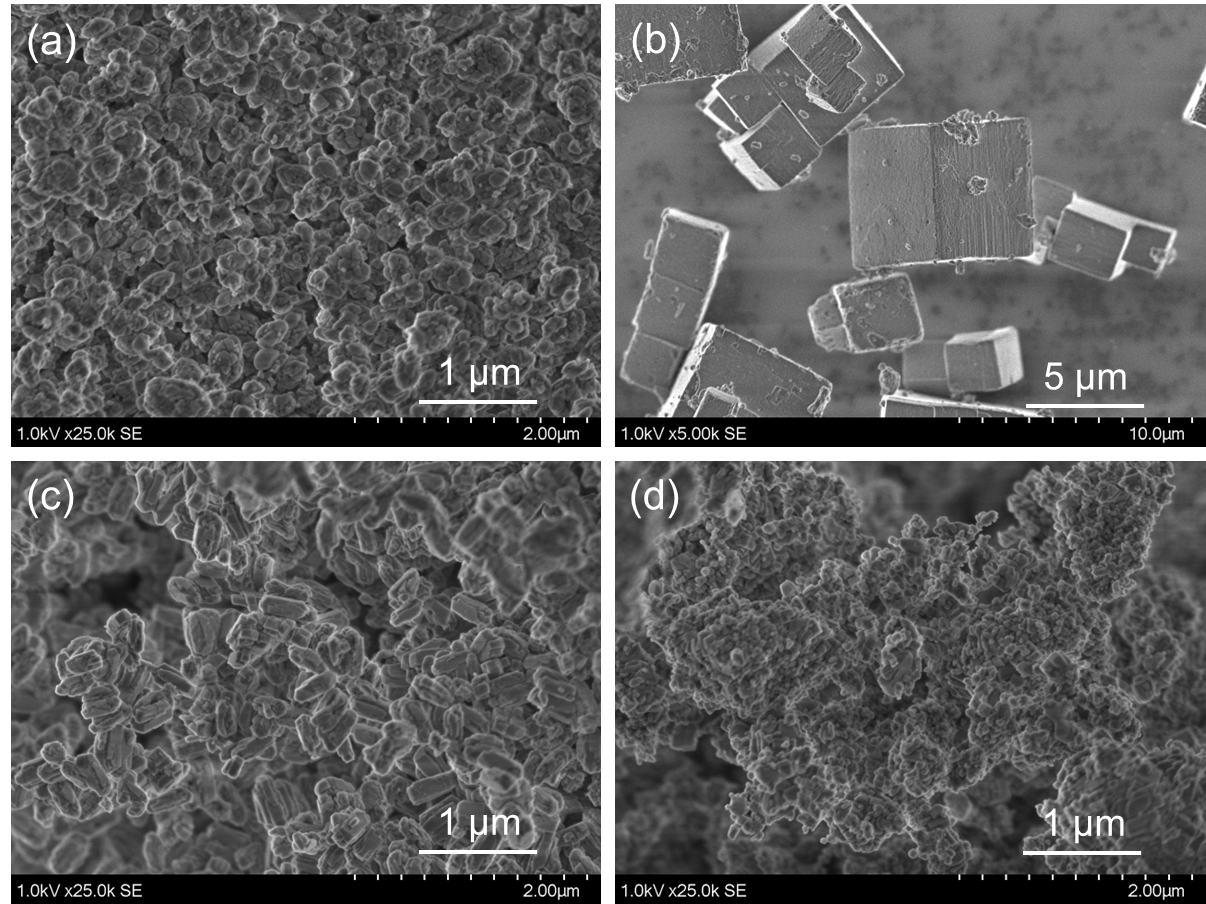


**Figure S2** SEM images of (a) SSZ-13, (b) SAPO-34, (c) ZSM-11, and (d) ZSM-5 zeolites.

**Figure S3** N_2_ adsorption and desorption isothermals of SSZ-13, SAPO-34, ZSM-11, and ZSM-5 zeolites.The isotherms for SAPO-34, ZSM-11, and ZSM-5 were offset vertically by 200, 400, and 600 cm^3^·g^-1^, respectively.


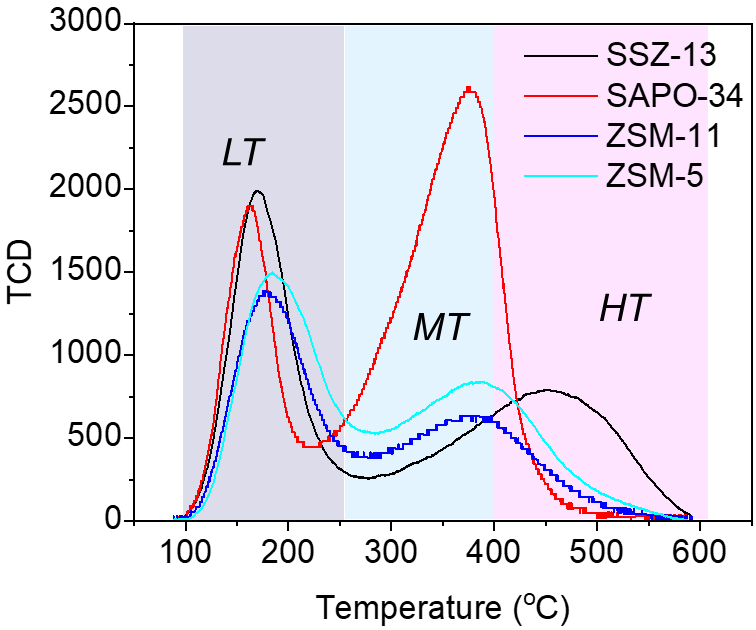


**Figure S4** NH_3_-TPD profiles of SSZ-13, SAPO-34, ZSM-11, and ZSM-5 zeolites.

**
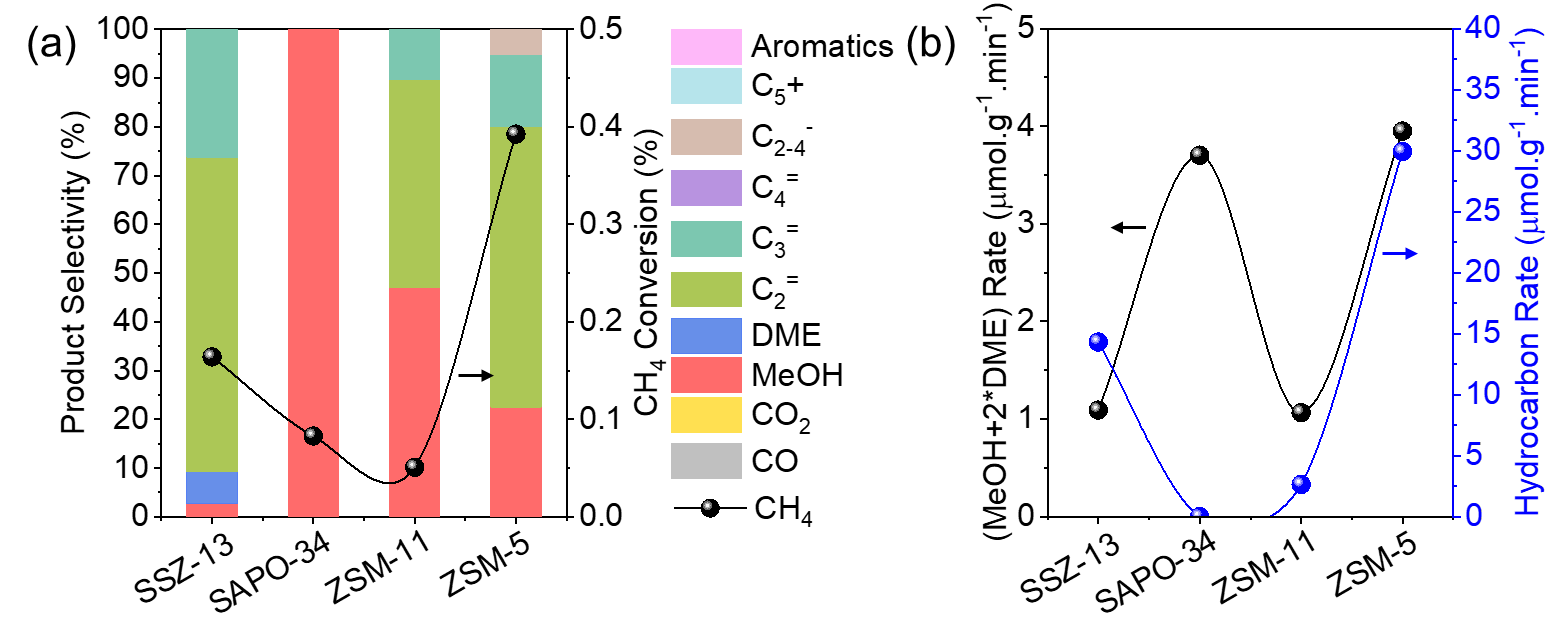
**

**Figure S5** Catalytic performance of transition-metal-free SSZ-13, SAPO-34, ZSM-11 and ZSM-5 zeolites. (a) CH_4_ conversion and product distribution, and (b) product formation rate. Reaction conditions: 350 ^o^C, atmospheric pressure, 100 mg transition-metal-free acidic zeolite, CH_4_/N_2_O/H_2_O/Ar=10/10/2/3 ml·min^-1^.


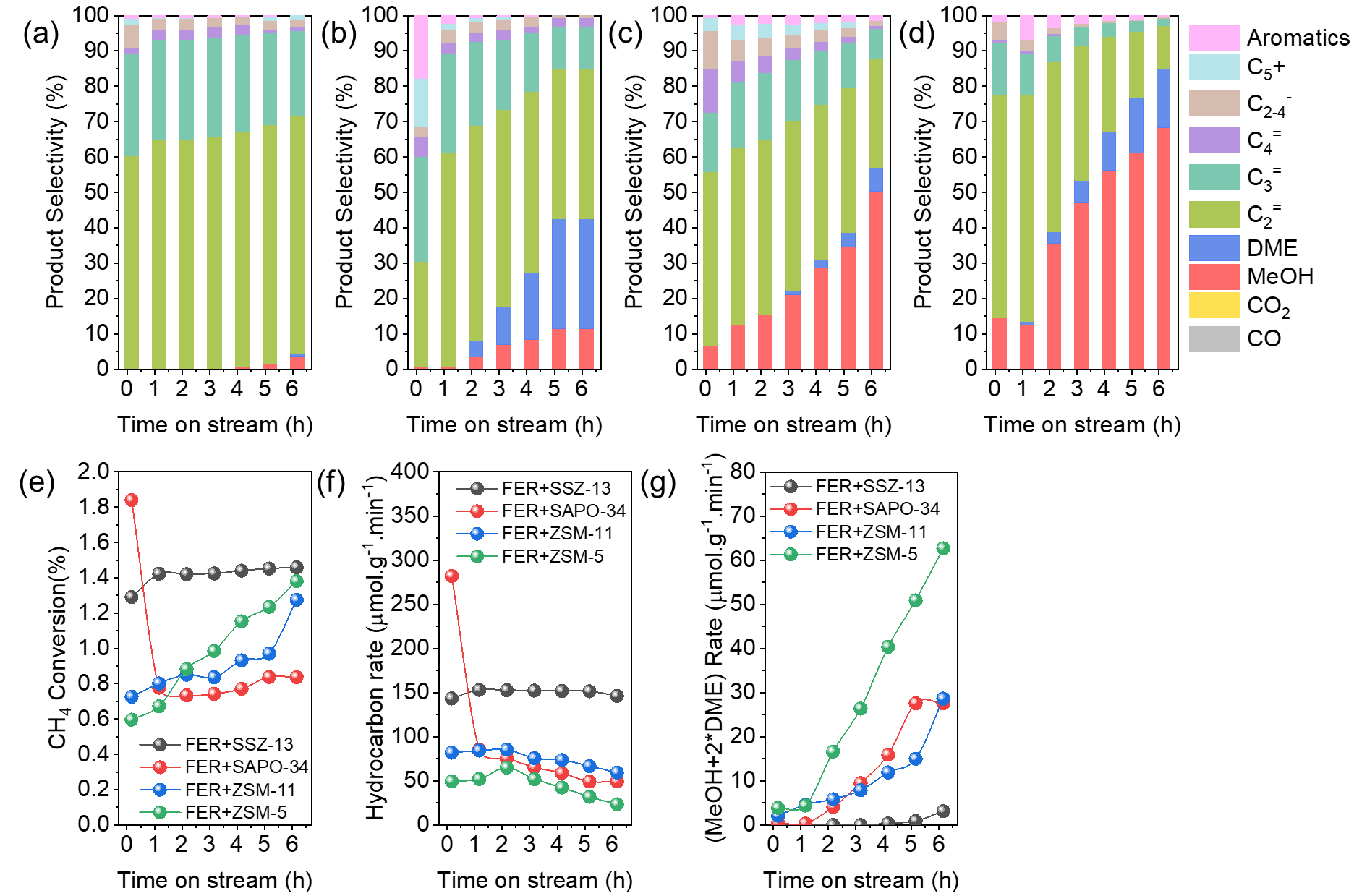


**Figure S6** Stability tests in methane conversion over FER/acidic zeolite cascades. Product distribution of (a) FER+ SSZ-13, (b) FER+ SAPO-34, (c) FER+ ZSM-11, (d) FER+ ZSM-5. Comparative analysis of (e) CH_4_ conversion, (f) hydrocarbon formation rate, and (g) (MeOH + 2*DME) formation rate. Reaction conditions: 350 ^o^C, atmospheric pressure, 50 mg FER, 50 mg acid zeolite, CH_4_/N_2_O/H_2_O/Ar=10/10/2/3 ml·min^-1^.


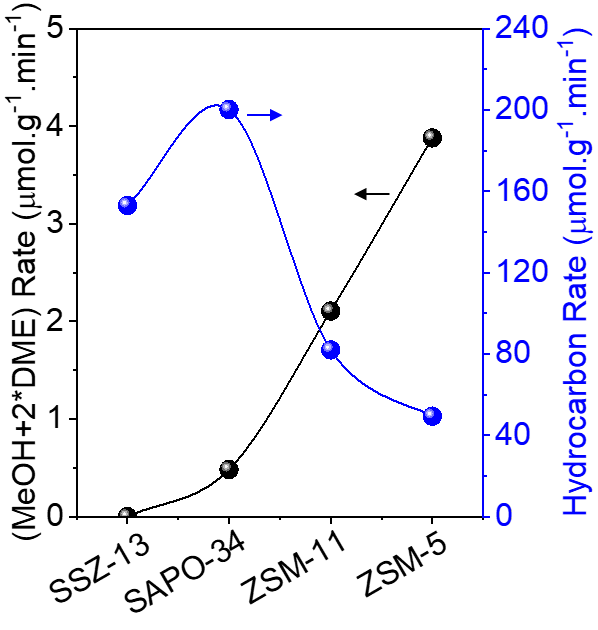


**Figure S7** Product formation rate over FER/acidic zeolite cascades with varied topologies. Reaction conditions: 350 ^o^C, 50 mg FER+50 mg acidic zeolite, CH_4_/N_2_O/H_2_O/Ar=10/10/2/3 ml·min^-1^, dual-bed mode, TOS=0.17 h.


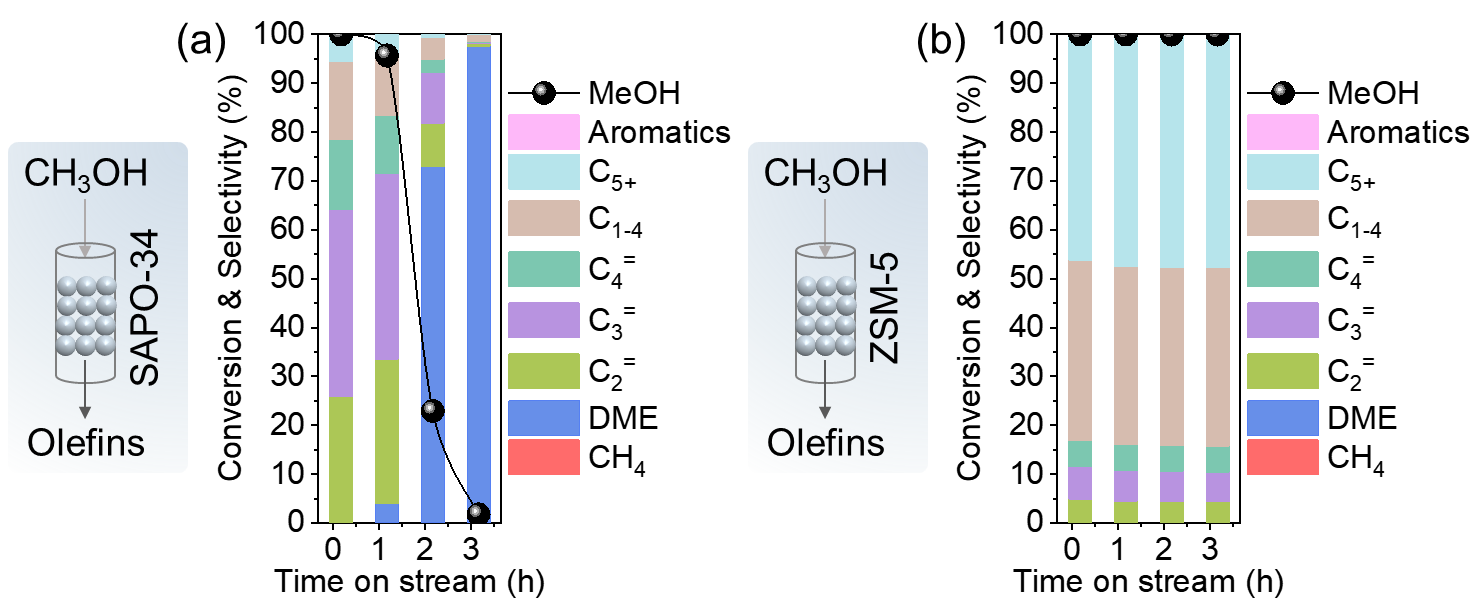


**Figure S8** Time courses of the methanol to hydrocarbon reaction at 350 °C on (a) SAPO-34 and (b) ZSM-5. Reaction condition: 100 mg catalyst, 10 vol% methanol in Ar gas, W/F_MeOH_ = 34 g·h·mol^-1^.

**
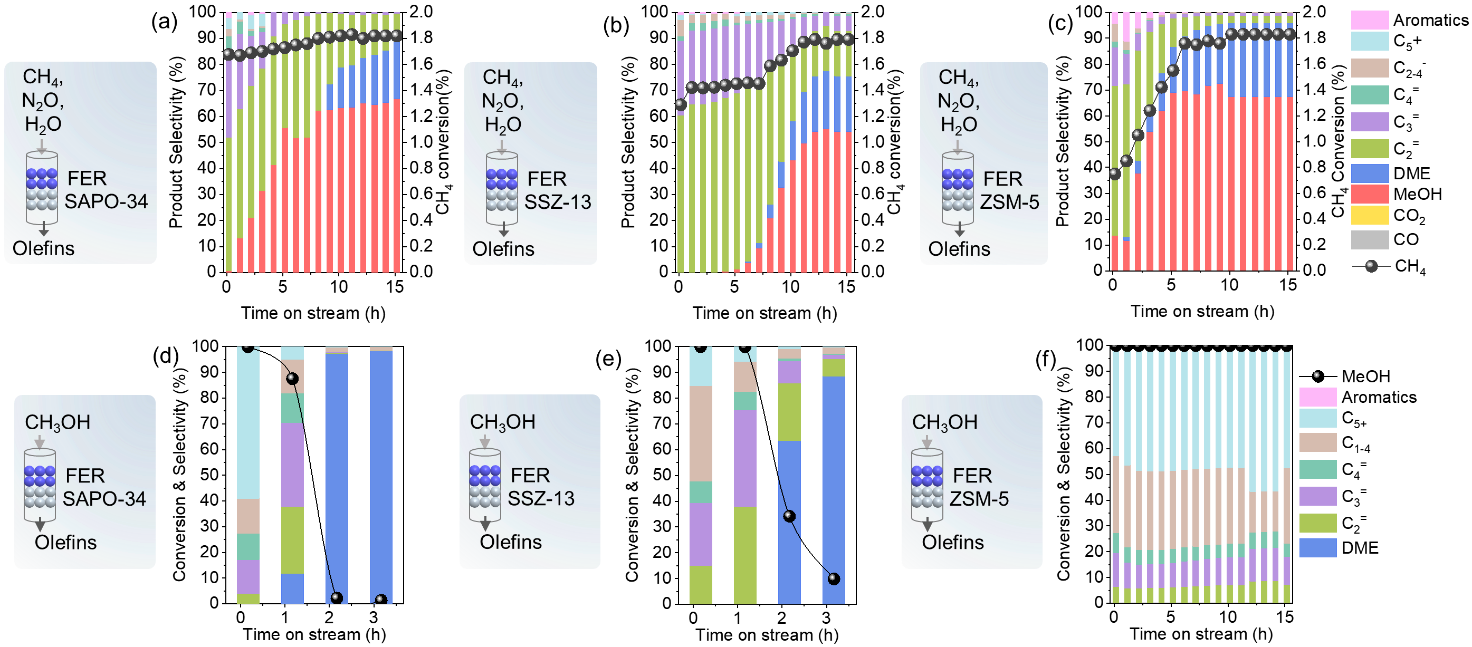
**

**Figure S9** Time courses of the methane to hydrocarbon reaction at 350 °C on (a) 50 mg FER +50 mg SAPO-34, (b) 50 mg FER +50 mg SSZ-13(10), (c) 50 mg FER +50 mg ZSM-5. Reaction condition: 100 mg catalyst, in dual-bed mode, CH_4_/N_2_O/H_2_O/Ar=10/10/2/3 ml·min^-1^. Time courses of the methanol to hydrocarbon reaction at 350 °C on (d) 50 mg FER +50 mg SAPO-34, (e) 50 mg FER +50 mg SSZ-13(10), (f) 50 mg FER +50 mg ZSM-5. Reaction condition: 100 mg catalyst, in dual-bed mode, 10 vol% methanol in Ar gas, W/F_MeOH_ = 34 g·h·mol^-1^.

**Figure S10** XRD patterns of SZZ-13 zeolites with varying gel Si/Al ratios (*x*=5-100).


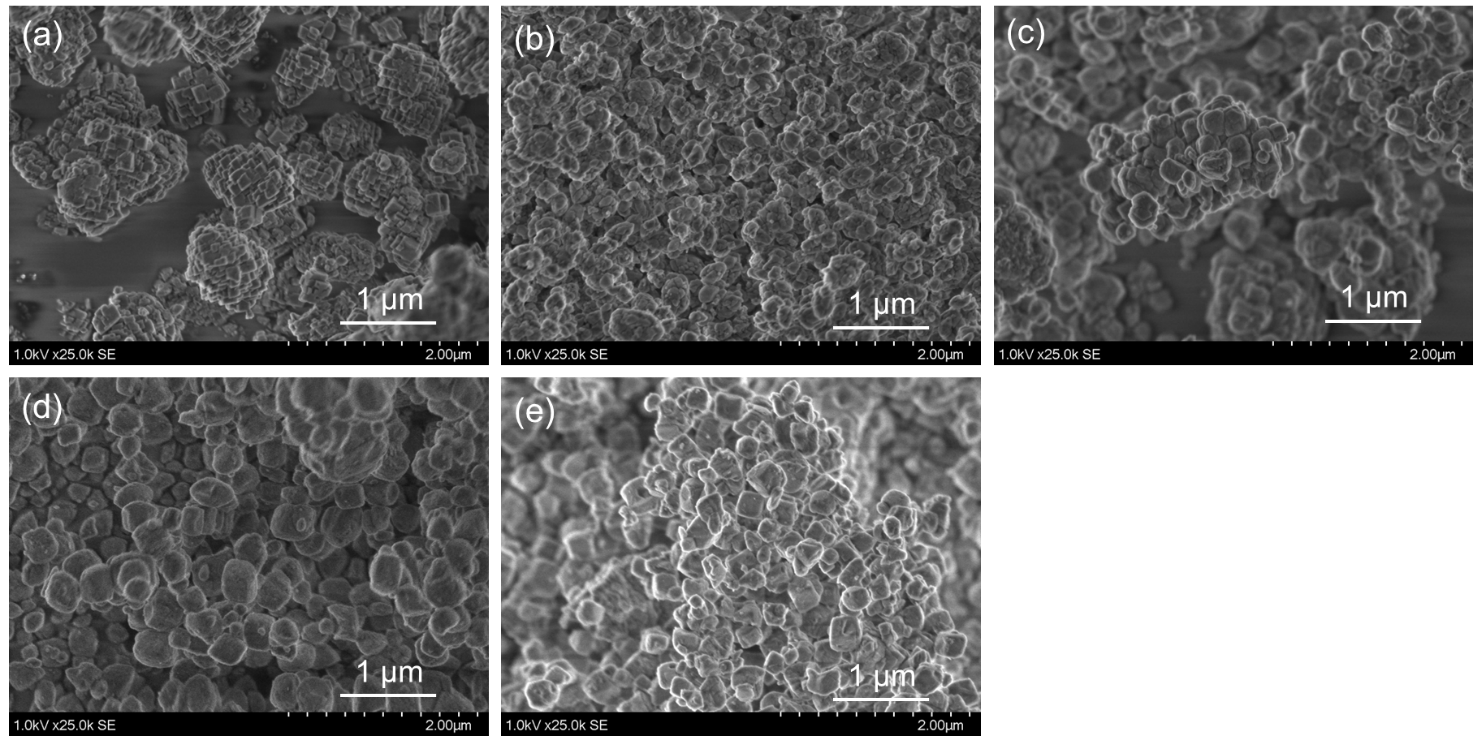


**Figure S11** SEM images of CHA zeolite with Si/Al ratio in the synthesis gel of (a) 5, (b) 10, (c) 25, (d) 50, and (e) 100.


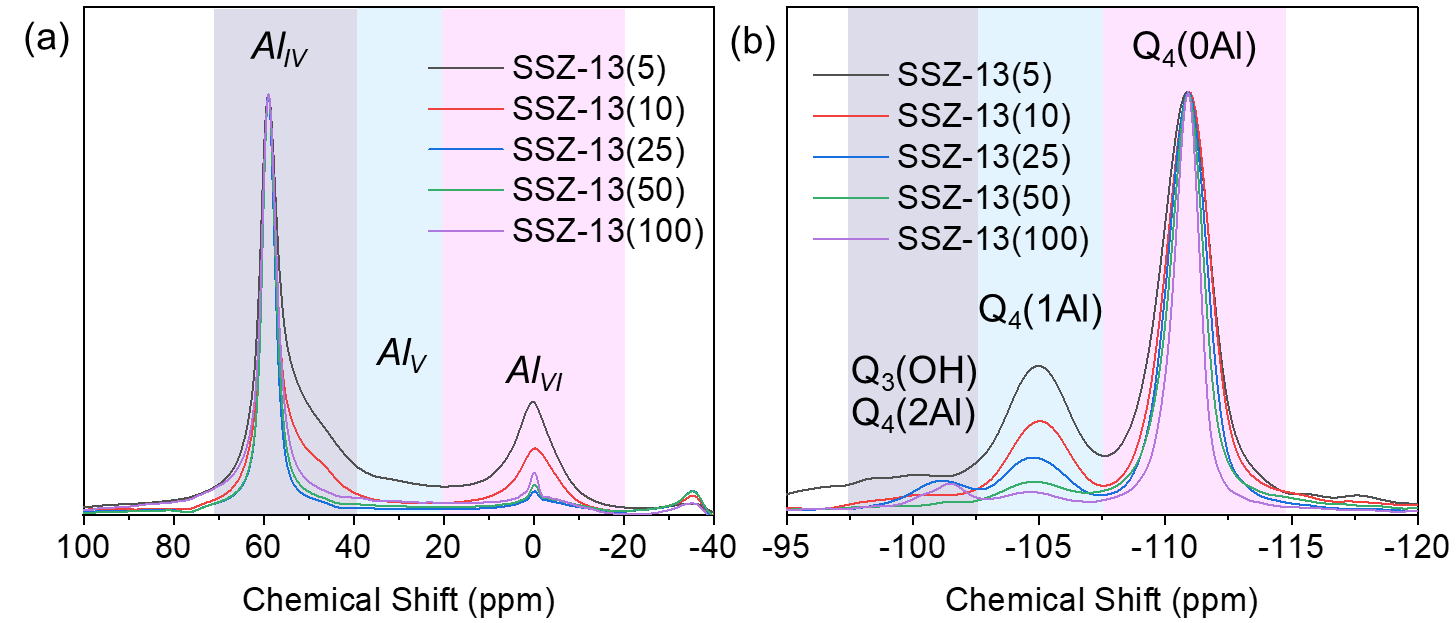
**Figure S12** (a) ^27^Al MAS NMR spectra and (b) ^29^Si MAS NMR spectra of SSZ-13(*x*), where *x* means the Si/Al ratio in the synthesis gel.


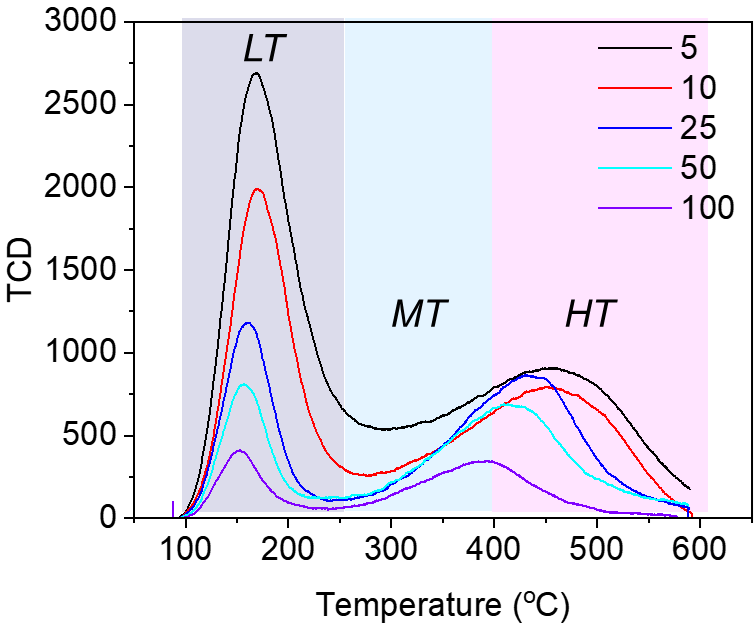


**Figure S13** NH_3_-TPD profiles of SSZ-13 zeolites varying gel Si/Al ratios (5-100).

**Figure S14** N_2_ adsorption and desorption isotherms of SSZ-13 zeolites varying gel Si/Al ratios (5-100). The isotherms for SSZ-13(10), SSZ-13(25), SSZ-13(50), and SSZ-13(100) were offset vertically by 200, 400, 600, and 800 cm^3^·g^-1^, respectively.


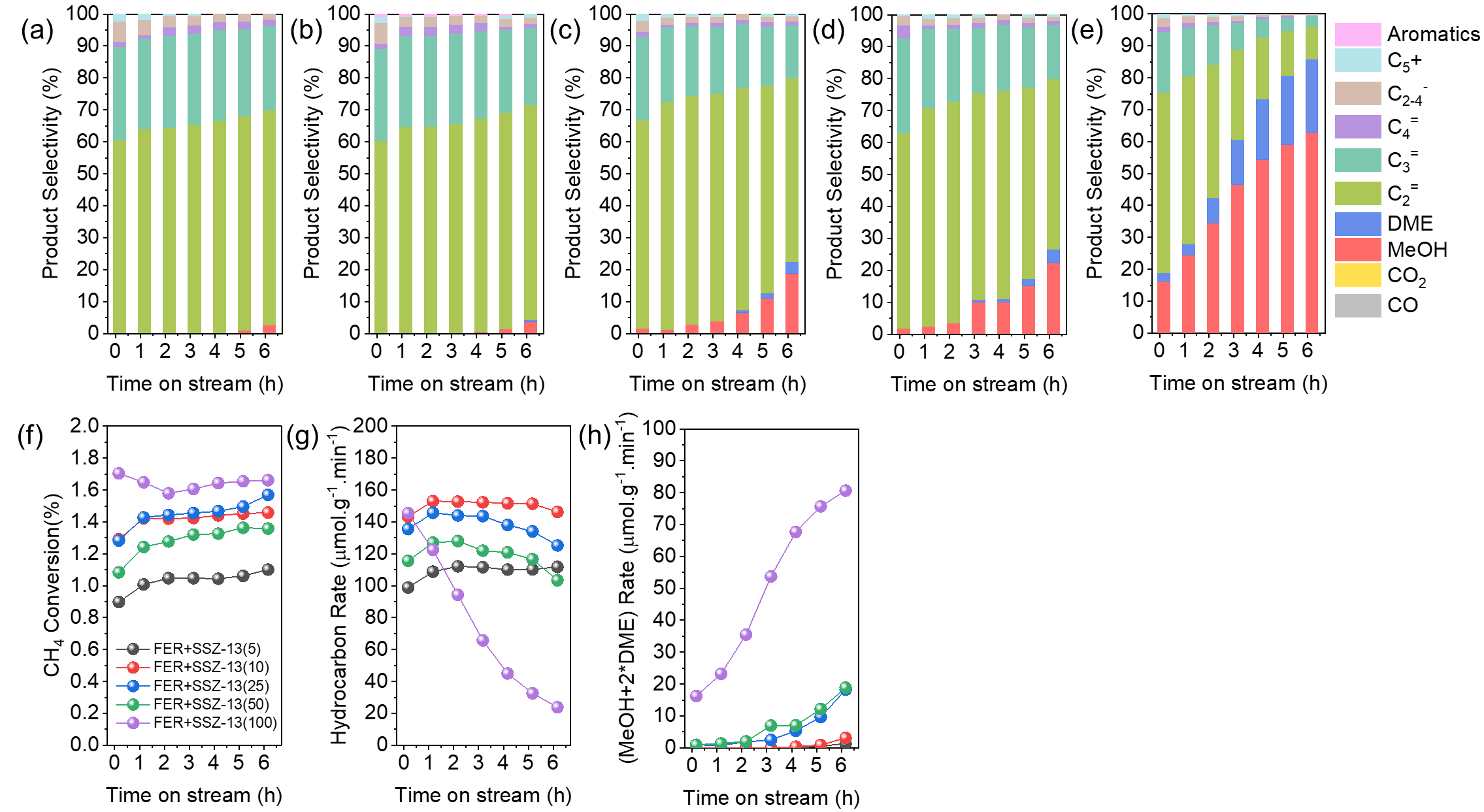


**Figure S15** Stability of FER/SSZ-13(x) cascades. Product distribution of (a) FER+ SSZ-13(5), (b) FER+ SSZ-13(10), (c) FER+ SSZ-13(25), (d) FER+ SSZ-13(50), (e) FER+ SSZ-13(100). Compare (f) CH_4_ conversion, (g) hydrocarbon formation rate, and (h) (MeOH + 2*DME) formation rate of tandem catalysts composed of FER and SSZ-13(x). Reaction conditions: 350 ^o^C, atmospheric pressure, 50 mg FER, 50 mg SSZ-13(x), CH_4_/N_2_O/H_2_O/Ar=10/10/2/3 ml·min^-1^.


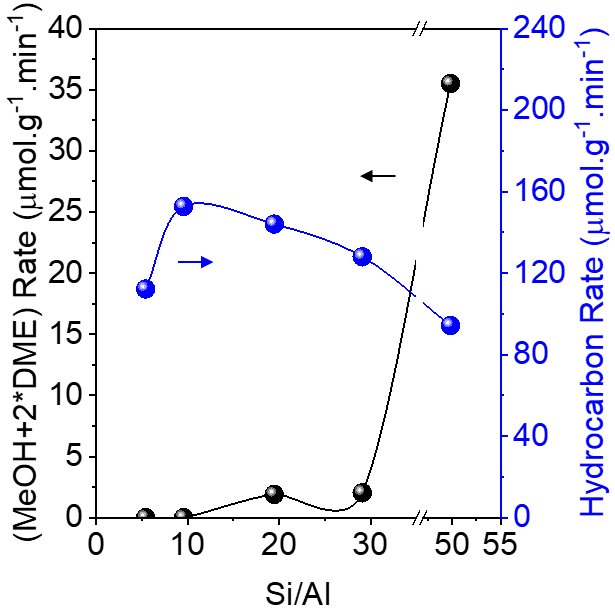


**Figure S16** Hydrocarbon formation rates over FER/SSZ-13 cascades with varied Si/Al ratios. Reaction conditions: 350 ^o^C, 50 mg FER, 50 mg acidic zeolite, CH_4_/N_2_O/H_2_O/Ar=10/10/2/3 ml·min^-1^, dual-bed mode, TOS=0.17 h.


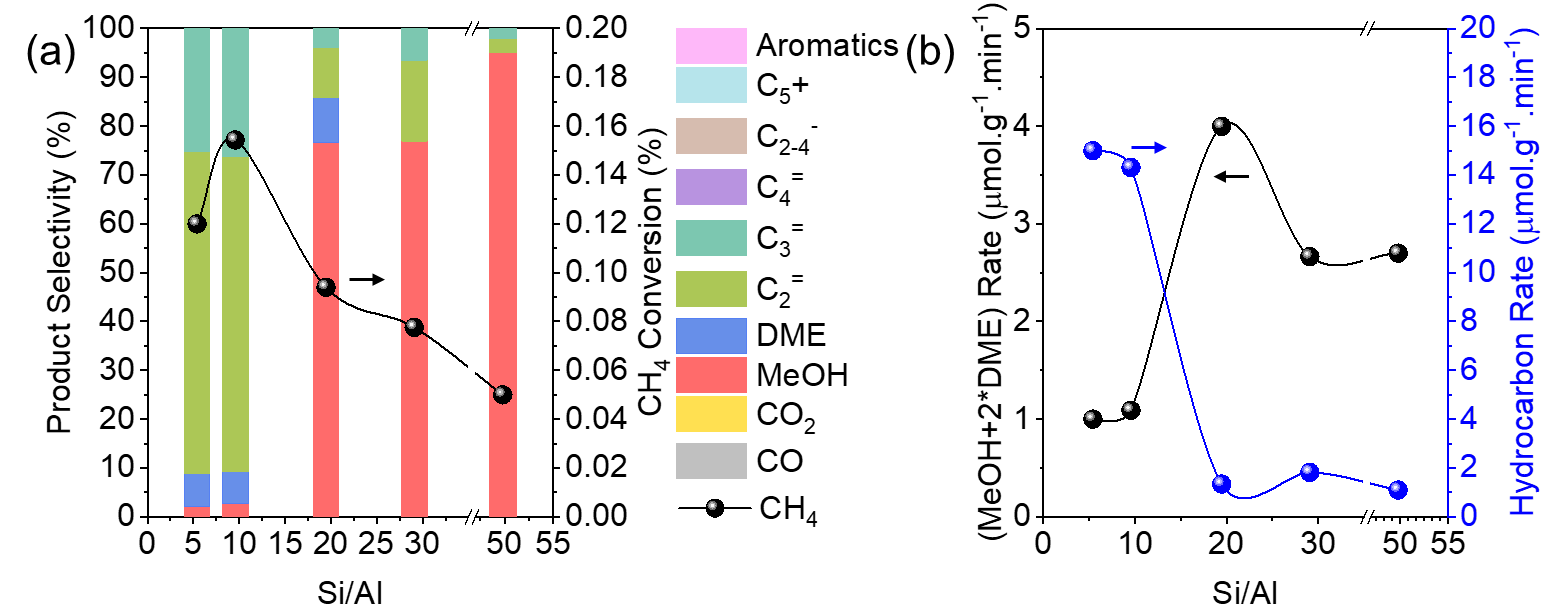


**Figure S17** Catalytic performance of H-SSZ-13(*x*) alone. (a) Product distribution and CH_4_ conversion and (b) product formation rate. Reaction conditions: 350 ^o^C, atmospheric pressure, 100 mg transition-metal-free SSZ-13 zeolite, CH_4_/N_2_O/H_2_O/Ar=10/10/2/3 ml·min^-1^.


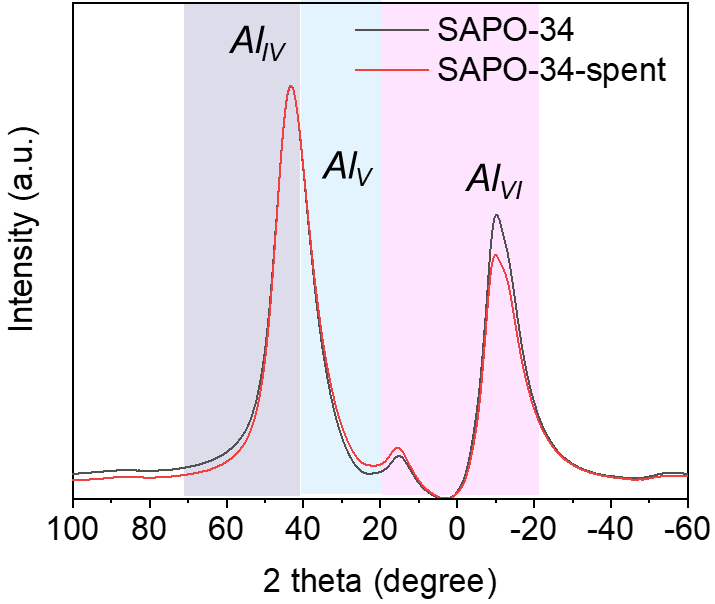


**Figure S18** Compare the ^27^Al MAS NMR spectra of the fresh and spent SAPO-34 zeolites.


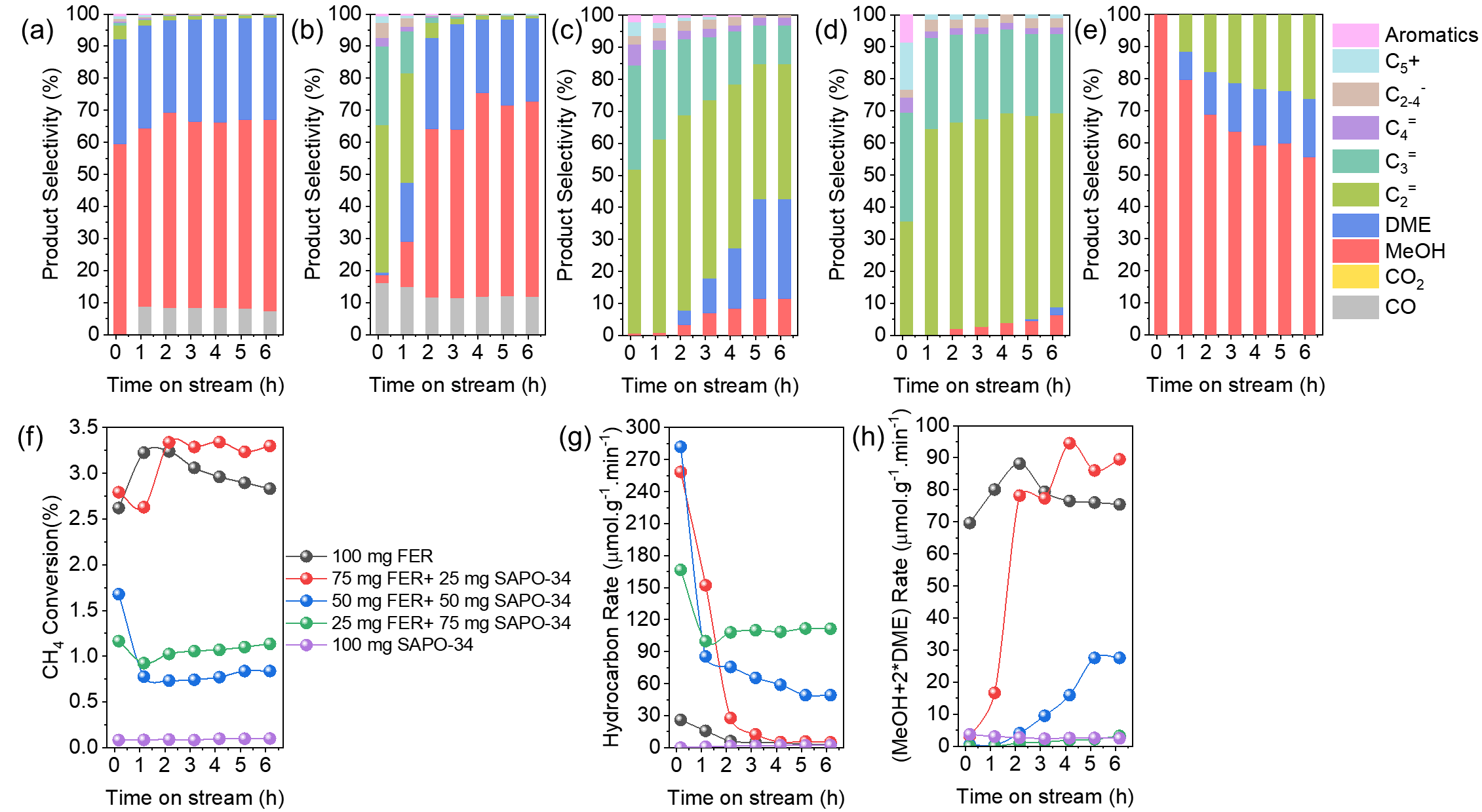


**Figure S19** Stability of FER/SAPO-34 cascades with varied mass ratios (total catalyst = 100 mg). Product distribution of (a) 100 mg FER, (b) 75 mg FER+ 25 mg SAPO-34, (c) 50 mg FER+ 50 mg SAPO-34, (d) 25 mg FER+ 75 mg SAPO-34, (e) 100 mg SAPO-34. Compare (f) CH_4_ conversion, (g) hydrocarbon formation rate, and (h) (MeOH+ 2*DME) formation rate of cascading different amounts of FER and SAPO-34 zeolites. Reaction conditions: 350 ^o^C, atmospheric pressure, *x* mg FER, (100-*x*) mg SAPO-34 zeolite, *x*=0~100, CH_4_/N_2_O/H_2_O/Ar=10/10/2/3 ml·min^-1^.


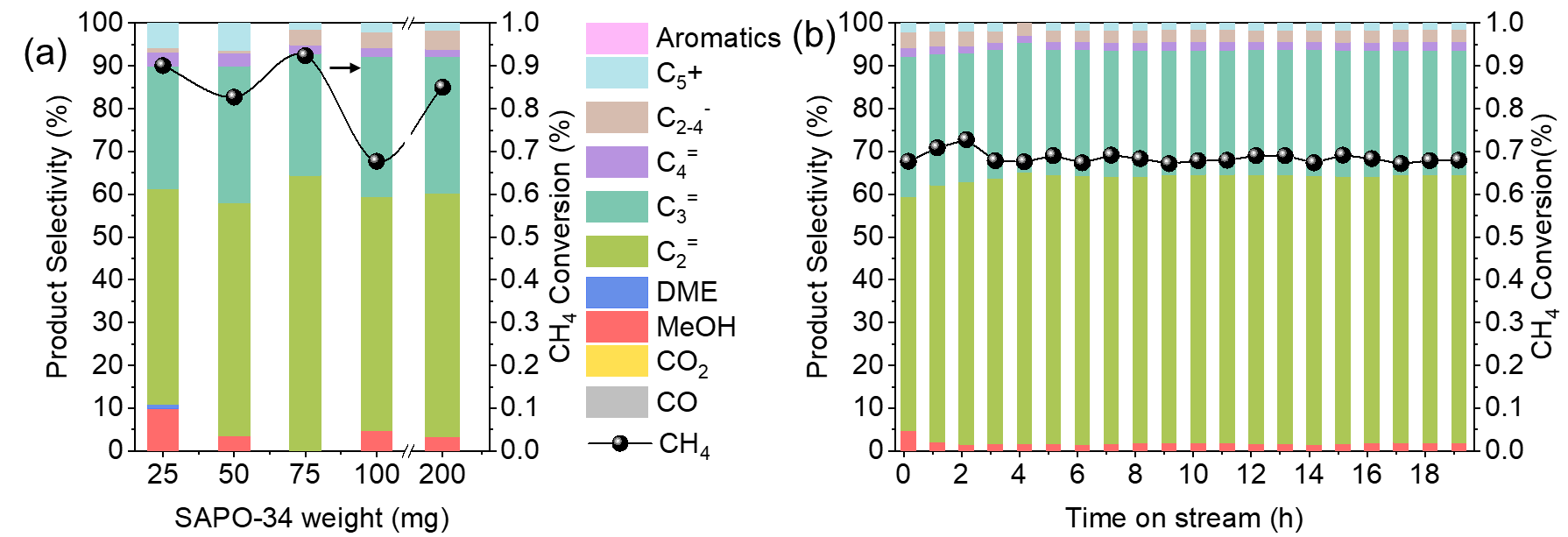


**Figure S20** (a) Product distribution and CH_4_ conversion with fixed FER (25 mg) and varied SAPO-34 mass (25-200 mg). Reaction conditions: 350 ^o^C, 25 mg FER, (25~200) mg SAPO-34 zeolite, CH_4_/N_2_O/H_2_O/Ar=10/10/2/3 ml·min^-1^. (b) Stability test of 25 mg FER + 100 mg SAPO-34. Reaction conditions: 350 ^o^C, 25 mg FER, 100 mg SAPO-34 zeolite, CH_4_/N_2_O/H_2_O/Ar=10/10/2/3 ml·min^-1^.


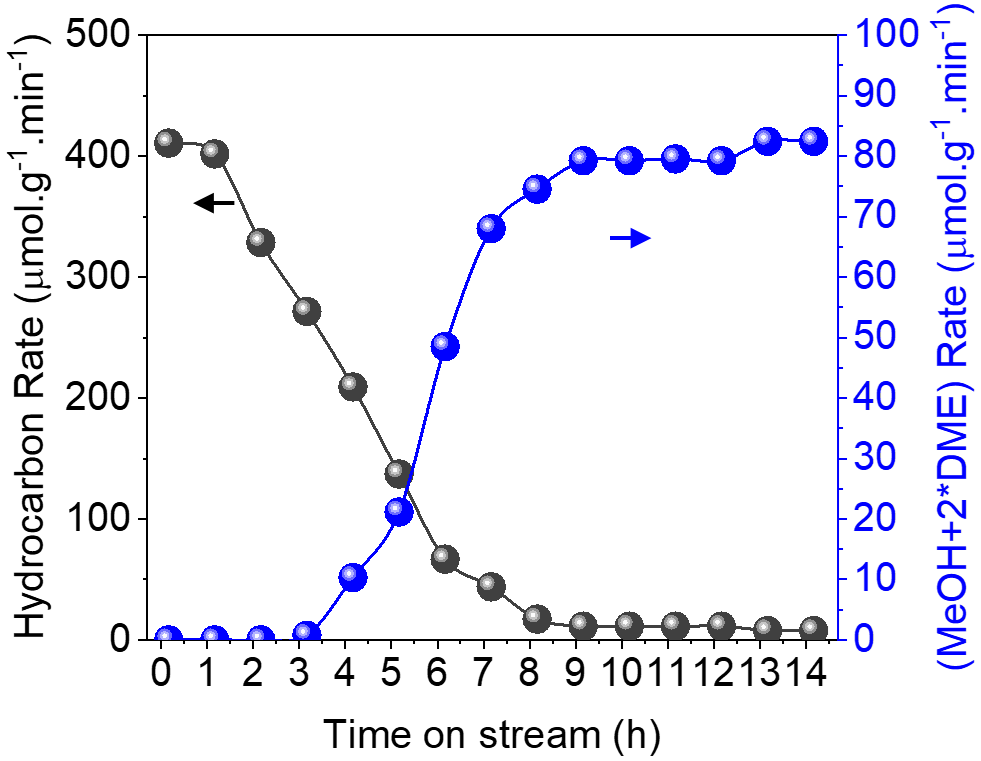


**Figure S21** Product formation rates during stability test of ultrasonically mixed FER/SAPO-34 (50:50 mg). Reaction condition: 50 mg FER, 50 mg SAPO-34, CH_4_/N_2_O/H_2_O/Ar=10/10/2/3 ml·min^-1^.


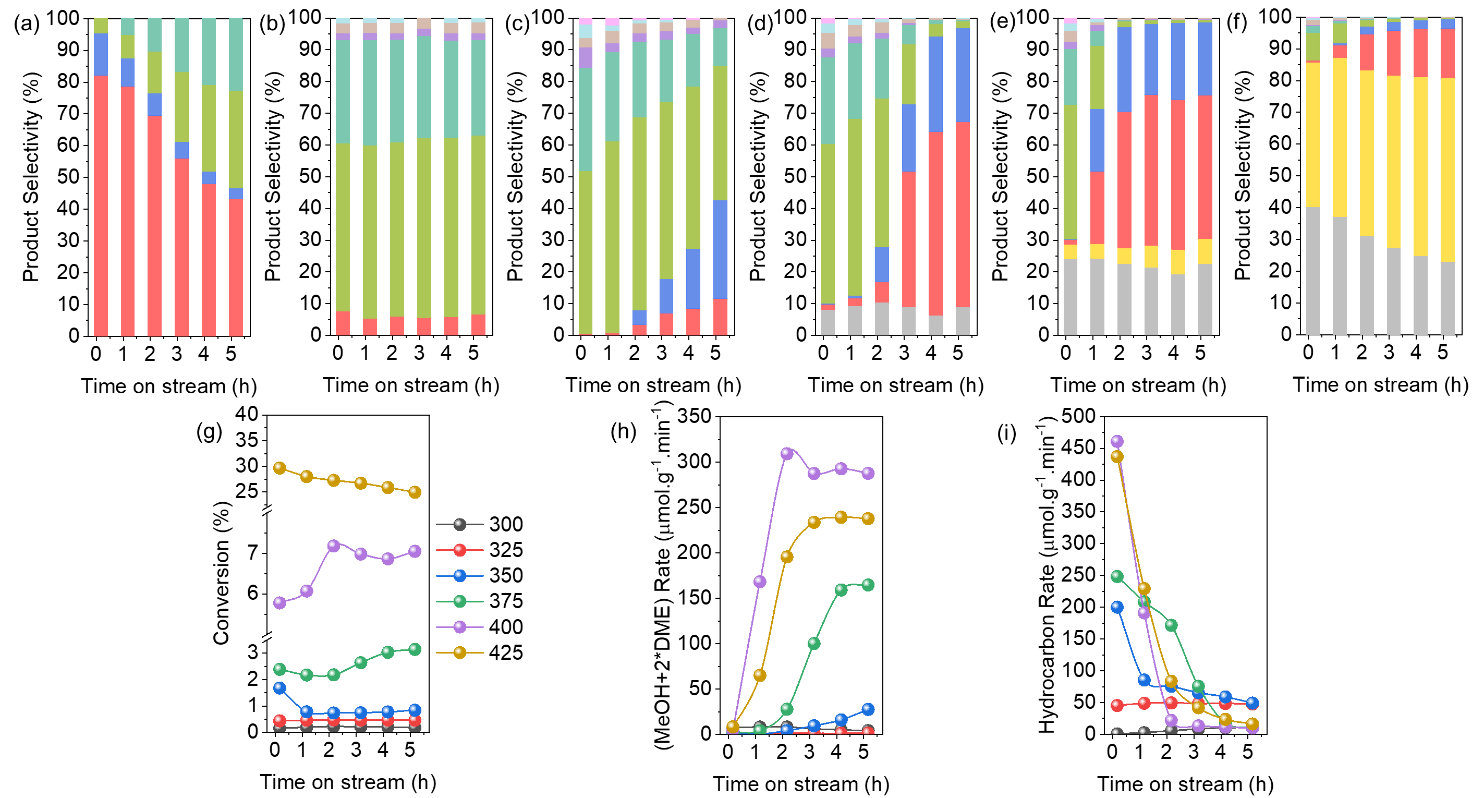


**Figure S22** Temperature-dependent performance of FER/SAPO-34 (50:50 mg). Product distribution of (a) 300, (b) 325, (c) 350, (d) 375, (e) 400, (f) 425 ^o^C. Compare (g) CH_4_ conversion, (h) (MeOH+ 2*DME) formation rate, and (i) hydrocarbon formation rate of cascading FER and SAPO-34 zeolite at different temperatures. Reaction conditions: 300-425 ^o^C, 50 mg FER, 50 mg SAPO-34, CH_4_/N_2_O/H_2_O/Ar=10/10/2/3 ml·min^-1^.

Table S1. Textual properties of zeolites.

| Sample | S_BET_(m^2^·g^-1^) ^a^ | V_Total_(cm^3^·g^-1^) ^a^ | S_EXT_(m^2^·g^-1^) ^b^ | V_Mic_(cm^3^·g^-1^) ^b^ |
| --- | --- | --- | --- | --- |
| H-FER | 329 | 0.30 | 33 | 0.11 |
| SSZ-13(SSZ-13(10)) | 689 | 0.81 | 80 | 0.27 |
| SAPO-34 | 574 | 0.27 | 3 | 0.26 |
| ZSM-11 | 400 | 0.51 | 50 | 0.16 |
| ZSM-5 | 376 | 0.37 | 92 | 0.12 |
| SSZ-13(5) | 682 | 0.48 | 55 | 0.29 |
| SSZ-13(10) | 689 | 0.81 | 80 | 0.27 |
| SSZ-13(25) | 783 | 0.43 | 28 | 0.34 |
| SSZ-13(50) | 660 | 0.46 | 25 | 0.28 |
| SSZ-13(100) | 695 | 0.47 | 34 | 0.30 |

*^a^* by the Brunauer–Emmett–Teller (BET) equation on the N_2_ adsorption isotherms.

*^b^* by the t-plot method based on the adsorption isotherms.

Table S2. Chemical composition and acid amount of zeolites.

| Sample | Si/Al *^a^* | Acid amount (mmol/g) *^b^* | | | | |
| --- | --- | --- | --- | --- | --- | --- |
|  |  | Weak | Medium | Strong | Total |  |
| H-FER | 28 | 0.39 | 0.62 |  | 1.01 |  |
| SSZ-13 | 10 | 0.44 | 0.18 | 0.74 | 1.36 |  |
| SAPO-34 | 0.05 | 0.38 | 1.27 |  | 1.65 |  |
| ZSM-11 | 25 | 0.25 | 0.63 |  | 0.88 |  |
| ZSM-5 | 21 | 0.49 | 0.63 |  | 1.12 |  |
| SSZ-13(5) | 5 | 0.55 | 0.35 | 0.79 | 1.69 |  |
| SSZ-13(10) | 10 | 0.44 | 0.18 | 0.74 | 1.36 |  |
| SSZ-13(25) | 20 | 0.24 | 0.28 | 0.28 | 0.8 |  |
| SSZ-13(50) | 30 | 0.14 | 0.29 | 0.18 | 0.61 |  |
| SSZ-13(100) | 50 | 0.06 | 0.10 | 0.11 | 0.27 |  |

*^a^* by ICP-AES.

*^b^* by NH_3_-TPD; the weak, medium, and strong acid amounts were fitted at approximately 150-250, 250-400, and 400-600 °C, respectively.

Table S3. Compare the reaction performance of composite zeolite catalysts.

| Sample | TOS  (h) | X_CH4_  (%) | S_CH3OH_(%) | S_DME_  (%) | S_C2=_  (%) | S_C3=_  (%) | S_C4=_  (%) | S_C2-4_ (%) | S_C5_  (%) | S_aromat_  (%) | r_MeOH+2*DME_  (μmol.g^-1^.min^-1^) | r_hydrocarb_  (μmol.g^-1^.min^-1^) |
| --- | --- | --- | --- | --- | --- | --- | --- | --- | --- | --- | --- | --- |
| FER/SSZ-13(10) | 0.16 | 1.3 | 0.0 | 0.0 | 60.4 | 28.7 | 1.8 | 6.4 | 1.8 | 0.9 | 0.0 | 143.4 |
|  | 1.16 | 1.4 | 0.0 | 0.0 | 64.9 | 28.3 | 2.9 | 3.1 | 0.2 | 0.6 | 0.0 | 153.0 |
|  | 2.16 | 1.4 | 0.0 | 0.0 | 64.9 | 28.3 | 2.9 | 3.1 | 0.2 | 0.6 | 0.0 | 152.7 |
|  | 3.16 | 1.4 | 0.0 | 0.0 | 65.7 | 28.2 | 2.9 | 2.6 | 0.0 | 0.6 | 0.0 | 152.3 |
|  | 4.16 | 1.4 | 0.6 | 0.0 | 66.7 | 27.4 | 2.6 | 2.3 | 0.0 | 0.4 | 0.4 | 151.7 |
|  | 5.16 | 1.5 | 1.4 | 0.0 | 67.6 | 26.1 | 1.1 | 2.4 | 1.1 | 0.3 | 0.9 | 151.5 |
|  | 6.17 | 1.5 | 3.7 | 0.6 | 67.3 | 24.2 | 1.2 | 2.0 | 1.1 | 0.0 | 3.2 | 146.2 |
| FER/SAPO-34 | 0.16 | 1.7 | 0.6 | 0.0 | 51.3 | 32.5 | 6.4 | 2.8 | 4.3 | 2.0 | 0.5 | 200.1 |
|  | 1.16 | 0.8 | 0.9 | 0.0 | 60.3 | 28.0 | 2.9 | 3.8 | 1.6 | 2.4 | 0.3 | 85.5 |
|  | 2.16 | 0.7 | 3.4 | 4.5 | 60.9 | 23.8 | 2.6 | 3.1 | 1.1 | 0.6 | 4.1 | 75.6 |
|  | 3.16 | 0.7 | 7.0 | 10.8 | 55.7 | 19.8 | 2.6 | 2.9 | 0.8 | 0.5 | 9.5 | 65.5 |
|  | 4.16 | 0.8 | 8.5 | 18.9 | 51.1 | 16.6 | 1.9 | 2.6 | 0.0 | 0.4 | 15.9 | 58.9 |
|  | 5.16 | 0.8 | 11.5 | 31.1 | 42.2 | 12.1 | 2.4 | 0.6 | 0.0 | 0.0 | 27.6 | 49.2 |
|  | 6.17 | 0.8 | 11.5 | 31.1 | 42.2 | 12.1 | 2.4 | 0.6 | 0.0 | 0.0 | 27.6 | 49.2 |
| FER/ZSM-11 | 0.16 | 0.7 | 6.5 | 0.0 | 49.3 | 16.7 | 12.5 | 10.6 | 3.9 | 0.5 | 2.1 | 82.0 |
|  | 1.16 | 0.8 | 12.8 | 0.0 | 50.0 | 18.4 | 6.0 | 5.9 | 4.2 | 2.7 | 4.6 | 84.3 |
|  | 2.16 | 0.9 | 15.5 | 0.0 | 49.4 | 19.1 | 4.7 | 5.1 | 4.1 | 2.2 | 5.9 | 85.4 |
|  | 3.16 | 0.8 | 21.1 | 1.3 | 47.9 | 17.3 | 3.2 | 3.9 | 2.9 | 2.4 | 7.9 | 75.7 |
|  | 4.16 | 0.9 | 28.6 | 2.5 | 43.7 | 15.3 | 2.5 | 3.2 | 2.0 | 2.1 | 11.9 | 73.6 |
|  | 5.16 | 1.0 | 34.7 | 4.1 | 41.0 | 12.8 | 1.5 | 2.5 | 1.9 | 1.6 | 15.0 | 66.7 |
|  | 6.17 | 1.3 | 50.3 | 6.6 | 31.0 | 8.3 | 1.0 | 1.5 | 0.0 | 1.3 | 28.6 | 59.4 |
| FER/ZSM-5 | 0.16 | 0.6 | 14.6 | 0.0 | 63.2 | 14.4 | 0.8 | 5.3 | 0.0 | 1.7 | 3.9 | 49.4 |
|  | 1.16 | 0.7 | 12.5 | 1.0 | 64.1 | 11.7 | 0.6 | 3.4 | 0.0 | 6.7 | 4.4 | 52.2 |
|  | 2.16 | 0.9 | 35.6 | 3.2 | 48.1 | 7.4 | 0.4 | 1.7 | 0.0 | 3.5 | 16.6 | 65.0 |
|  | 3.16 | 1.0 | 47.0 | 6.5 | 38.1 | 5.2 | 0.0 | 0.8 | 0.0 | 2.3 | 26.4 | 52.1 |
|  | 4.16 | 1.2 | 56.3 | 11.0 | 26.8 | 3.9 | 0.0 | 0.6 | 0.0 | 1.4 | 40.4 | 42.4 |
|  | 5.16 | 1.2 | 61.2 | 15.6 | 18.8 | 3.0 | 0.0 | 0.5 | 0.0 | 0.9 | 50.9 | 31.9 |
|  | 6.17 | 1.4 | 68.4 | 16.7 | 12.2 | 2.1 | 0.0 | 0.4 | 0.0 | 0.4 | 62.7 | 23.4 |
| FER/SSZ-13(5) | 0.16 | 0.9 | 0.0 | 0.0 | 60.5 | 29.3 | 1.6 | 6.3 | 2.2 | 0.0 | 0.0 | 98.9 |
|  | 1.16 | 1.0 | 0.0 | 0.0 | 64.0 | 28.3 | 1.2 | 4.7 | 1.8 | 0.0 | 0.0 | 108.8 |
|  | 2.16 | 1.0 | 0.0 | 0.0 | 64.5 | 28.8 | 2.7 | 3.4 | 0.6 | 0.0 | 0.0 | 112.2 |
|  | 3.16 | 1.0 | 0.0 | 0.0 | 65.5 | 28.4 | 2.6 | 3.0 | 0.5 | 0.0 | 0.0 | 111.6 |
|  | 4.16 | 1.0 | 0.0 | 0.0 | 66.7 | 28.4 | 2.5 | 2.4 | 0.0 | 0.0 | 0.0 | 110.2 |
|  | 5.16 | 1.1 | 1.2 | 0.0 | 66.9 | 27.5 | 2.3 | 2.2 | 0.0 | 0.0 | 0.6 | 110.3 |
|  | 6.17 | 1.1 | 2.7 | 0.0 | 67.2 | 26.4 | 2.1 | 1.7 | 0.0 | 0.0 | 1.3 | 111.8 |
| FER/SSZ-13(25) | 0.16 | 1.4 | 1.6 | 0.0 | 65.3 | 26.0 | 1.4 | 3.6 | 2.0 | 0.0 | 0.9 | 135.5 |
|  | 1.16 | 1.4 | 1.3 | 0.0 | 71.2 | 23.3 | 1.0 | 2.3 | 0.9 | 0.0 | 0.9 | 145.7 |
|  | 2.16 | 1.5 | 3.0 | 0.0 | 71.5 | 21.8 | 1.0 | 1.8 | 0.9 | 0.0 | 1.9 | 144.0 |
|  | 3.16 | 1.5 | 3.9 | 0.0 | 71.2 | 21.1 | 1.1 | 1.8 | 0.9 | 0.0 | 2.5 | 143.6 |
|  | 4.16 | 1.5 | 6.5 | 0.8 | 69.5 | 20.1 | 1.3 | 1.7 | 0.0 | 0.0 | 5.3 | 138.1 |
|  | 5.16 | 1.6 | 11.1 | 1.7 | 65.0 | 18.4 | 1.4 | 1.6 | 0.9 | 0.0 | 9.7 | 134.0 |
|  | 6.17 | 1.4 | 18.9 | 3.6 | 57.6 | 16.5 | 1.3 | 1.5 | 0.7 | 0.0 | 18.3 | 125.2 |
| FER/SSZ-13(50) | 0.16 | 1.1 | 2.0 | 0.0 | 61.0 | 29.9 | 4.0 | 2.8 | 0.4 | 0.0 | 1.0 | 115.6 |
|  | 1.16 | 1.2 | 2.5 | 0.0 | 68.2 | 24.9 | 1.1 | 2.2 | 1.1 | 0.0 | 1.4 | 127.0 |
|  | 2.16 | 1.3 | 3.6 | 0.0 | 69.3 | 22.9 | 1.3 | 1.9 | 1.0 | 0.0 | 2.0 | 128.0 |
|  | 3.16 | 1.3 | 10.0 | 0.9 | 64.6 | 20.4 | 1.5 | 1.6 | 0.9 | 0.0 | 7.0 | 122.0 |
|  | 4.16 | 1.3 | 10.1 | 0.9 | 65.2 | 20.6 | 1.5 | 1.6 | 0.0 | 0.0 | 7.1 | 120.9 |
|  | 5.16 | 1.4 | 15.1 | 2.4 | 59.8 | 18.9 | 1.4 | 1.6 | 0.8 | 0.0 | 12.1 | 116.7 |
|  | 6.17 | 1.4 | 22.2 | 4.5 | 53.2 | 17.0 | 1.3 | 1.2 | 0.7 | 0.0 | 18.9 | 103.5 |
| FER/SSZ-13(100) | 0.16 | 1.7 | 16.3 | 2.5 | 56.6 | 19.0 | 1.7 | 2.8 | 1.1 | 0.0 | 16.3 | 145.4 |
|  | 1.16 | 1.6 | 24.4 | 3.6 | 52.6 | 15.4 | 1.3 | 2.1 | 0.7 | 0.0 | 23.2 | 122.4 |
|  | 2.16 | 1.6 | 34.4 | 7.9 | 41.8 | 12.3 | 1.0 | 1.7 | 0.8 | 0.0 | 35.5 | 94.3 |
|  | 3.16 | 1.6 | 46.6 | 14.2 | 28.1 | 8.5 | 0.7 | 1.3 | 0.7 | 0.0 | 53.7 | 65.8 |
|  | 4.16 | 1.6 | 54.4 | 18.9 | 19.3 | 5.9 | 0.7 | 0.7 | 0.0 | 0.0 | 67.7 | 45.0 |
|  | 5.16 | 1.7 | 59.1 | 21.7 | 13.9 | 4.3 | 0.7 | 0.4 | 0.0 | 0.0 | 75.7 | 32.5 |
|  | 6.17 | 1.7 | 62.9 | 22.9 | 10.4 | 3.2 | 0.3 | 0.4 | 0.0 | 0.0 | 80.6 | 23.8 |

**Reference**

[1] P. Xiao, L. Wang, H. Toyoda, Y. Wang, K. Nakamura, J. Huang, R. Osuga, M. Nishibori, H. Gies, T. Yokoi, *J Am Chem Soc* **2024**, *146*, 31969-31981.

[2] Y. Shen, T. T. Le, D. Fu, J. E. Schmidt, M. Filez, B. M. Weckhuysen, J. D. Rimer, *ACS Catalysis* **2018**, *8*, 11042-11053.

[3] T. Biligetu, Y. Wang, T. Nishitoba, R. Otomo, S. Park, H. Mochizuki, J. N. Kondo, T. Tatsumi, T. Yokoi, *Journal of Catalysis* **2017**, *353*, 1-10.
